# Mitochondrial proteomics of the acetic acid - induced programmed cell death response in a highly tolerant *Zygosaccharomyces bailii* - derived hybrid strain 

**DOI:** 10.15698/mic2016.02.477

**Published:** 2016-01-22

**Authors:** Joana F. Guerreiro, Belém Sampaio-Marques, Renata Soares, Ana V. Coelho, Cecília Leão, Paula Ludovico, Isabel Sá-Correia

**Affiliations:** 1 Institute for Bioengineering and Biosciences, Department of Bioengineering, Instituto Superior Técnico, Universidade de Lisboa, 1049-001 Lisbon, Portugal.; 2 Life and Health Sciences Research Institute (ICVS), School of Health Sciences, University of Minho, Braga 4710-057, Portugal.; 3 ICVS/3B’s - PT Government Associate Laboratory, Braga/Guimarães, Portugal.; 4 Instituto de Tecnologia Química e Biológica António Xavier, Universidade Nova de Lisboa, 2780-157 Oeiras, Portugal.

**Keywords:** yeast programmed cell death, Zygosaccharomyces bailii, acetic acid response, quantitative proteomics, mitochondrial proteomics

## Abstract

Very high concentrations of acetic acid at low pH induce programmed cell death (PCD) in both the experimental model *Saccharomyces cerevisiae* and in *Zygosaccharomyces bailii*, the latter being considered the most problematic acidic food spoilage yeast due to its remarkable intrinsic resistance to this food preservative. However, while the mechanisms underlying *S. cerevisiae* PCD induced by acetic acid have been previously examined, the corresponding molecular players remain largely unknown in *Z. bailii*. Also, the reason why acetic acid concentrations known to be necrotic for *S. cerevisiae* induce PCD with an apoptotic phenotype in *Z. bailii* remains to be elucidated. In this study, a 2-DE-based expression mitochondrial proteomic analysis was explored to obtain new insights into the mechanisms involved in PCD in the *Z. bailii* derived hybrid strain ISA1307. This allowed the quantitative assessment of expression of protein species derived from each of the parental strains, with special emphasis on the processes taking place in the mitochondria known to play a key role in acetic acid - induced PCD. A marked decrease in the content of proteins involved in mitochondrial metabolism, in particular, in respiratory metabolism (Cor1, Rip1, Lpd1, Lat1 and Pdb1), with a concomitant increase in the abundance of proteins involved in fermentation (Pdc1, Ald4, Dld3) was registered. Other differentially expressed identified proteins also suggest the involvement of the oxidative stress response, protein translation, amino acid and nucleotide metabolism, among other processes, in the PCD response. Overall, the results strengthen the emerging concept of the importance of metabolic regulation of yeast PCD.

## INTRODUCTION

Acetic acid is a commonly used anti-microbial additive, with a broad application in the preservation of acidic foods and beverages. However, under these conditions many yeasts and moulds are able to adapt and proliferate, becoming the prevailing contaminants of foods and beverages preserved at low pH 1,2. Among food spoilage yeasts, *Zygosaccharomyces bailii* is considered the most problematic, particularly in acidic foods, mainly due to its very high tolerance to carboxylic acids used as fungistatic preservatives 3,4,5. Elucidating the mechanisms underlying weak acid cytotoxicity that may affect cell viability, ultimately leading to cell death, is crucial to guide the improvement of food and beverage preservation practices, with the consequent minimization of the associated economic losses. Acetic acid is known to induce programmed cell death (PCD) in both the experimental model *Saccharomyces cerevisiae* 6 and in *Z. bailii* 7. However, this effect is observed at much higher concentrations of the acid for *Z. bailii, *in the range of 320 - 800 mM, compared to 20 - 120 mM for *S. cerevisiae* 6,7,8. Depending on the acid concentration, acetic acid may induce a PCD either with an apoptotic or a necrotic phenotype 6,7,8.

In *S. cerevisiae*, acetic acid - induced PCD with an apoptotic phenotype is known to be mediated by mitochondria, an organelle that fulfills crucial functions and hosts a range of signaling, metabolic and energetic pathways, involved in regulation of cell death and differentiation, processes implicated in numerous human diseases 9,10. In yeast, this mitochondria - dependent PCD process displays the most common apoptotic hallmarks, such as translocation of pro-apoptotic factors (e.g. cytochrome *c*) to the cytosol and mitochondrial reactive oxygen species (ROS) production and accumulation (reviewed in 11,12). Furthermore, the mechanisms underlying acetic acid - induced *S. cerevisiae *PCD have been previously characterized through proteome-wide analyses of *S. cerevisiae* response to a pro-apoptotic concentration of acetic acid 13,14. These analyses implicated the general amino-acid control (GAAC) system, further shown to be associated with a severe intracellular amino-acid starvation, as well as the TOR pathway in acetic acid - induced death 13 and the role of the metacaspase Yca1 in acetic acid - induced death 14. A genome-wide analysis also highlighted the importance of several carbohydrate metabolic processes, mitochondrial function, protein synthesis and modification, amino acid metabolism, oxidative stress response, protein phosphorylation and histone deacetylation for increased or decreased resistance to acetic acid - induced PCD in *S. cerevisiae *15. Similarly, in* Z. bailii*, acetic acid also induces PCD with an apoptotic phenotype characterized by plasma membrane integrity preservation, the occurrence of DNA fragmentation and mitochondrial ultrastructural changes, specifically, decrease of the cristae number, formation of myelinic bodies and swelling, accompanied by mitochondrial depolarization, while preserving mitochondrial membrane integrity 7. However, the molecular players involved in this process remain largely unknown.

Previously, a quantitative two-dimensional electrophoresis (2-DE)-based expression proteomics approach was used to analyze for the first time the proteomic response to sub-lethal concentrations of acetic acid in the *Z. bailii* derived hybrid strain ISA1307, isolated from a sparkling wine plant 16, highlighting several molecular mechanisms that underlie the global adaptive response in this highly acetic acid tolerant strain 17. This strain was first considered to belong to the *Z. bailii* species but was later shown to be an interspecies hybrid between *Z. bailii* and an unidentified closely related species 18. For years, this strain has been used in the study of *Z. bailii* physiology, since it exhibits many traits of interest, in particular its high tolerance to acetic acid 16,17,19. In order to extend the analysis of this strain response to lethal concentrations of acetic acid, in particular at the level of the mitochondria that is known to play a key role in acetic acid - induced PCD in *S. cerevisiae *13 and to suffer extensive ultrastructural changes during the PCD process in *Z. bailii* 7, we performed in this study a 2-DE-based expression proteomic analysis, following sub-fractionation of yeast mitochondrial proteins 5. The use of the 2-DE-based quantitative proteomic analysis, combined with sub-cellular fractionation, allowed us to focus our analysis specifically on mitochondrial proteins, mitochondrial functions, mitochondrial controlled processes and PCD 20. In addition, while the previous study on the proteomic response to sub-lethal concentrations of acetic acid was severely limited by the lack of the genome sequence of the hybrid strain examined 17, the analysis here presented benefited from this knowledge and the careful annotation of the genome 18, to identify the majority of proteins with altered content in acetic acid challenged cells.

## RESULTS

### Characterization of strain ISA1307 global response to an acetic acid concentration inducing PCD

**Figure 1:**
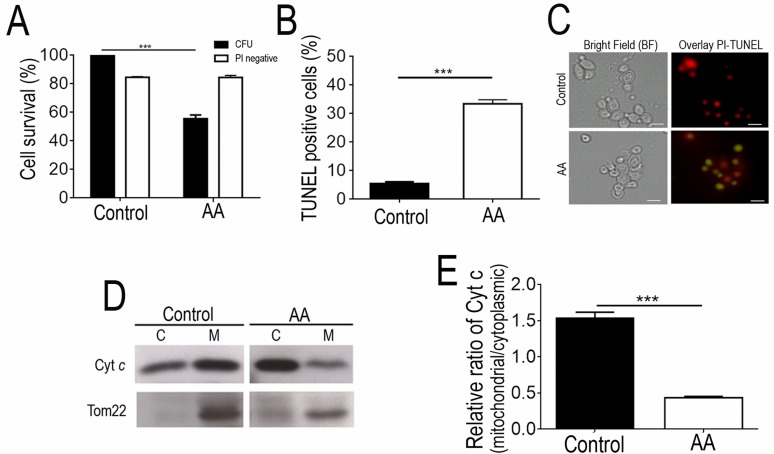
FIGURE 1: Analysis of PCD markers in strain ISA1307 acetic acid - treated cells prior to mitochondrial proteomic analysis. **(A) **Comparison of the survival rate evaluated by colony-forming unit (CFU) counts and propidium iodide (PI) exclusion of strain ISA1307 cells upon acetic acid (AA) treatment with a equitoxic dose of acetic acid, during 200 min. **(B)** Percentage of cells and **(C)** epifluorescence and bright field micrographs of untreated and acetic acid (AA)-treated strain ISA1307 cells displaying TUNEL positive phenotype. Cells were co-stained with propidium iodide in order to facilitate nuclei visualization. Bar, 5 µm. **(D)** Immunoblot analysis of cytochrome *c* (Cyt. *c*) and Tom22 protein levels in 20 µg of cytoplasmic (C) and mitochondrial (M) protein extracts of 300 mM acetic acid - treated and untreated strain ISA1307 cells. **(E)** Densitometric analysis of cyt. *c* protein levels. Analysis was performed using the ratio of cyt. *c* levels between the mitochondrial and the cytoplasmic protein extracts of three independent biological replicates. Bands were quantified in Quantity One® software. Significance of the data was determined by two-way ANOVA (***P < 0.001).

Although it has already been reported that acetic acid concentrations in the range of 320 - 800 mM induce a PCD process in *Z. bailii* hybrid strain ISA1307 7, this study was started by validating the experimental conditions to be used and to confirm the occurrence of massive PCD under the specific scale-up conditions of acetic acid treatment required to isolate higher amounts of mitochondrial protein extracts suitable to carry out proteomic analysis. Our results demonstrated that under these settings, described in the Materials and Methods section, cellular viability dropped by about 50% (Fig. 1A) and these cells were found to commit to a PCD with an apoptotic phenotype. This was evidenced by the high number of propidium iodide (PI)-negative cells, indicating that plasma membrane integrity was preserved and no extensive necrosis has occurred (Fig. 1A), and by the detection of TUNEL-positive cells, indicating occurrence of DNA strand breaks typical of apoptotic phenotypes (Fig. 1A-C). Consistently, exposure to the acetic acid stressing conditions tested led to the translocation of cytochrome *c* from the mitochondria to the cytosol (Fig. 1D-E), similar to what has been previously described in *S. cerevisiae *3, further validating these experimental conditions for additional assays.

### Alterations in strain ISA1307 mitochondrial proteome in response to an acetic acid concentration inducing PCD

**Figure 2:**
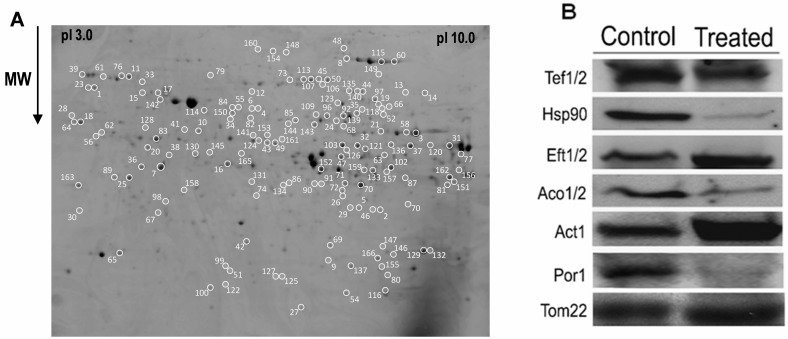
FIGURE 2: Reference map of strain ISA1307 mitochondrial proteome and immunoblot analysis of proteins found to be differentially expressed in 2-DE gels of ISA1307 acetic acid - treated cells. **(A)** The 2-DE PAGE-based protein reference map was generated from strain ISA1307 cells treated with an apoptosis - inducing acetic acid concentration. Spots altered upon treatment are represented by numbers. **(B) **Immunoblot analysis of the mitochondrial protein levels of Tef1/2, HSP90, Eft1/2, Aco1/2, actin, porin and the mitochondrial marker protein Tom22 in 20 µg of total protein extracts of strain ISA1307 cells treated with 300 mM of acetic acid for 200 min or untreated. The anti-HSP90 antibody detects both protein species of the HSP90 chaperone family, Hsc82 and Hsp82.

In order to obtain new insights into the mechanisms behind PCD induced by acetic acid in *Z. bailii* hybrid strain ISA1307, particularly the role that mitochondria play in the process, a 2-DE-based expression proteomic analysis was carried out. The methodology previously developed to allow examination of *Z. bailii *cytosolic proteome 17 was coupled to a prefractionation step for mitochondria isolation and proteins in this mitochondrial fraction were separated by 2-DE. Approximately 1300 spots within a pI range between 3 and 10, and a molecular mass between 18 and 95 kDa were separated and detected in the 2-DE gels obtained. A reference map representative of all the gels obtained was prepared (Fig. 2A).

The quantitative alterations occurring in the proteome of cells challenged with the death - inducing concentration of acetic acid tested led to the identification of 158 protein spots as having a different abundance in cells exposed to the acid compared with control cells. Of these, 139 spots were identified (corresponding to 87 unique proteins), 71 of which exhibited a decreased abundance, and 68 an increased abundance in the acetic acid challenged cell population (Table S1). For this identification by MS, the information recently made available through the sequencing and annotation of this strain’s genome 18 was explored. To validate part of the results obtained by 2-DE, a Western blot analysis was used to determine the abundance of some of the proteins identified (Tef1/Tef2, Hsp90, Eft1/Eft2, Aco1/Aco2, Act1 and Por1), as well as the mitochondrial marker protein Tom22 (Fig. 2B).

The efficiency of the prefractionation step carried out for mitochondria isolation, made prior to 2-DE, was confirmed based on the predicted subcellular localization of each of the 139 *Z. bailii *hybrid strain proteins (Table S1). This localization was predicted according to the information available in SGD (http://www.yeastgenome.org) database for the corresponding *S. cerevisiae *homologue proteins since very little or no information is available for the *Z. bailii *hybrid strain under study and much of the information used in the genome annotation 18 has been based on homology analysis with other species, including *S. cerevisiae*. The results obtained indicate that the prefractionation step was quite efficient, since about 60% of the proteins obtained have already been described as being located in the *S. cerevisiae *mitochondria (Table S1). Although this value is below the one previously reported for the crude mitochondrial fraction 21, it still represents a reasonable enrichment of mitochondrial proteins. The remaining 40% of the proteins are essentially cytoplasmic and/or nuclear proteins. However, some of those proteins (Tkl1, Pdc1, Ssb1, Sam2 and Spe3) have previously been identified in other mitochondrial proteomic analyses 22,23 or are predicted to interact with mitochondrial proteins (e.g., Pdc1, Shm2, Cdc19) according to the interaction network between proteins obtained from STRING (http://string.embl.de/) 24 (Fig. S1). This suggests that they may not be contaminants, but instead have a yet unidentified mitochondrial localization or strong interaction with mitochondrial proteins, an hypothesis further strengthened by the fact that the abundance of the mitochondrial marker protein remains unaltered in the different conditions analyzed (Fig. 2B).

**Figure 3:**
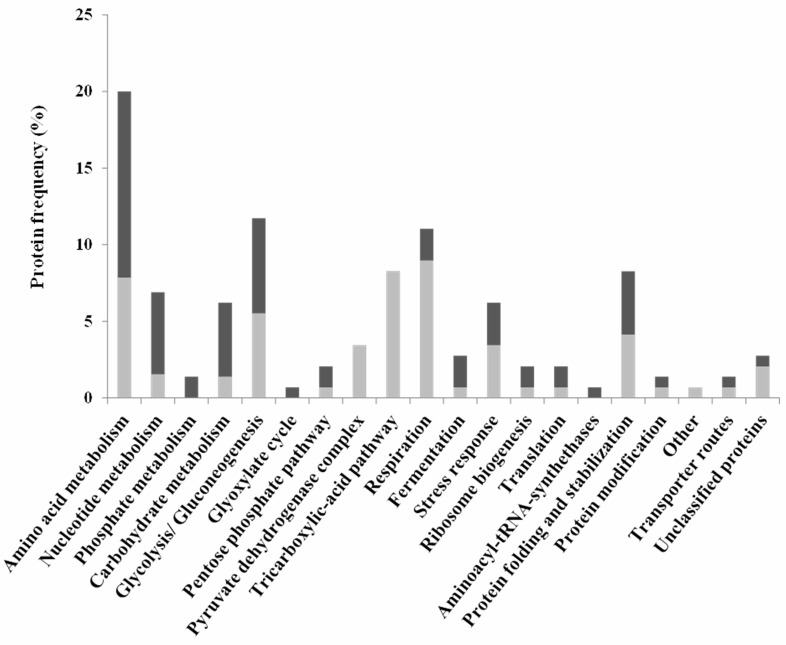
FIGURE 3: Clustering, based on physiological function, of ISA1307 proteins whose abundance changes in response to PCD - inducing concentrations of acetic acid. The proteins whose abundance was found to change in ISA1307 cell population exposed to acetic acid - inducing PCD concentration, according to the results obtained in the quantitative proteomic analysis, were clustered using the MIPS functional catalogue. The frequency in the dataset of proteins whose abundance was increased (black bars) or decreased (light grey bars) under acetic acid - induced PCD is also indicated.

The proteins whose relative abundance was found to be different during growth in the presence or absence of acetic acid were clustered into functional groups using the MIPS functional catalogue (http://mips.helmholtz-muenchen.de/funcatDB/) and SGD (http://www.yeastgenome.org) database (Table S1). The results obtained are summarized in figure 3 and highlight the contribution of cellular metabolism, in particular carbohydrate and energetic metabolic processes such as amino acid and nucleotide metabolism, stress response, protein synthesis and fate, among other processes, in the PCD response in this *Z. bailii *- derived hybrid strain. In addition, changes in abundance of the protein species derived from each of the two parental strains (*Z. bailii *and a yet unidentified species) were in general coordinated upon acetic acid - induced PCD (the only notable exceptions being Pgk1 and Adh3) (Table S1 and Table S2) 18. The main responses found to occur in the proteome of acetic acid challenged cells are described in detail in the following section.

### Energy and carbohydrate metabolism 

**Figure 4:**
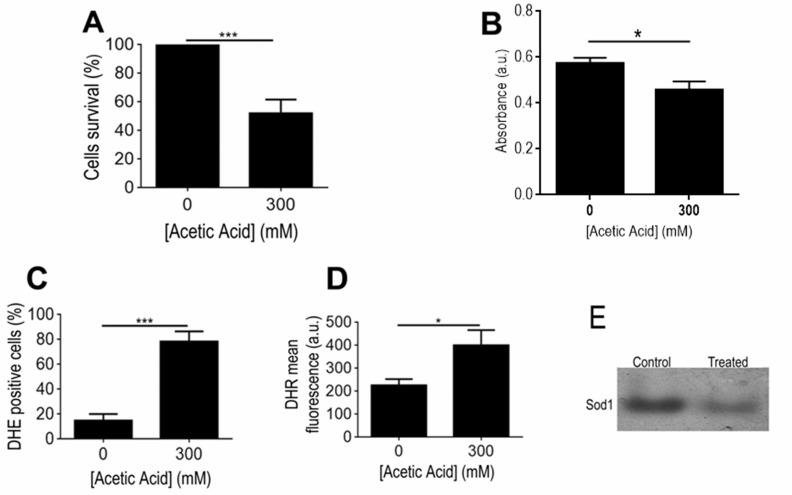
FIGURE 4: Determination of respiratory activity, ROS and superoxide dismutase activity in strain ISA1307 upon acetic acid treatment. **(A)** Comparison of the survival rate of strain ISA1307 cells treated with 300 mM of acetic acid. **(B) **Absorbance measurements (480 nm) of formed formazan using the artificial electron acceptor INT (2-p-iodophenyl-3-p-nitrophenyl-5-phenyl tetrazolium chloride) in ISA1307 cells. Bar graphs indicate mean and standard error of the mean (SEM) absorbance (arbitrary units) measured in 2 OD equivalents/sample in six independent experiments. **(C)**, **(D)** FACS measurements of superoxide anions using the probe dihydrioethidium and hydrogen peroxide using the probe dihydrorhodamine 123 (DHR) in ISA1307 cells. **(E) ***In situ *determination of superoxide dismutase activities in 300 mM acetic acid - treated and untreated (control) ISA1307 cells. Bar graphs indicate mean and standard error of the mean (SEM) fluorescence/cell (arbitrary units) measured in 25,000 cells/sample in three independent experiments. Significance of the data was determined by two-way ANOVA (*P < 0.05; **P < 0.01; ***P < 0.001).

Some of the main responses found to occur in the mitochondrial proteome of acetic acid challenged ISA1307 cells involved the alteration in the protein content of several proteins linked to energy metabolism. Significantly, there was a marked decrease in the content of proteins involved in the TCA cycle (Aco1, Aco2, Kgd2, Mdh1, Idh2 and Cit1), respiration and oxidative phosphorylation, including two proteins of the bc1 complex (Cor1, Rip1) and several subunits of the mitochondrial ATP synthase (Atp2, Atp7), as well as in proteins belonging to the pyruvate dehydrogenase complex (Lpd1, Lat1 and Pdb1), which catalyzes the oxidative decarboxylation of pyruvate. Since these results were suggestive of a general decrease of mitochondrial metabolism in the cell population exposed to acetic acid, we used an adapted INT - based assay in order to determine the proportion of respiring ISA1307 cells in both populations, based on their capacity to reduce INT to its corresponding formazan, as previously described for *S. cerevisiae* 25. As expected, the results obtained demonstrate that the respiratory capacity of the cell population under acetic acid - induced PCD was indeed decreased when compared with the control population (Fig. 4B).

Consistent with a decreased mitochondrial metabolism, the abundance of a variety of enzymes shared by the glycolytic and neoglucogenic pathways (Fba1, Cdc19, Hxk2, Tpi1, Tdh2, Pgk1 and Eno1) exhibited an altered content in acetic acid challenged cells with about half of the protein species identified having increased content, while the other half had decreased content in these cells (Table S1, Fig. 3). Additionally, the abundance of the tranketolase Tkl1, involved in the pentose phosphate pathway (PPP), malate synthase (Mls1), an enzyme of the glyoxylate cycle, and of a mitochondrial aldehyde dehydrogenase (Ald4), required for growth on ethanol, also exhibited increased levels (Table S1). This is in contrast with what has been previously described to happen in *S. cerevisiae *where cells *en route* to acetic acid - induced PCD exhibited a general decrease in glycolytic enzymes and a shift towards the PPP 14. The same holds true in what concerns pyruvate decarboxylase enzyme (Pdc1), known to be involved in pyruvate fermentation to acetaldehyde and ethanol, found to be decreased in *S. cerevisiae *14 but having increased content in strain ISA1307 upon acetic acid - induced PCD (Table S1). An increase in the content of the D-lactate dehydrogenase (Dld3), involved in lactate catabolism, was also observed (Table S1). A higher abundance was also registered for some proteins known to be involved in the metabolism of energy reserves, such as glycogen or trehalose (Bmh1, Ugp1), or in the metabolism of glycerol (Gut2) while a protein involved in glycerol synthesis (Gpd1) also showed decreased content upon acetic acid - induced PCD (Table S1). Interestingly, Bmh1 has been previously suggested to be implicated in *S. cerevisiae *PCD 26. However, in contrast to what had been observed in *S. cerevisiae *cells under acetic acid - induced PCD 14, its content increased in ISA1307 cells suffering acetic acid - induced PCD.

### Stress response

Consistent with the increased accumulation of ROS, namely of superoxide anion and hydrogen peroxide observed in yeast cells upon treatment with a lethal concentration of acetic acid (Fig. 4C-D), several proteins involved in cellular response to oxidative stress showed altered abundance under those conditions. In particular, the mitochondrial superoxide dismutase Sod2, involved in the conversion of superoxide into hydrogen peroxide, the cytoplasmic thioredoxin peroxidase Tsa1, which degrades hydrogen peroxide, and the nitric oxide oxidoredutase Yhb1, involved in nitric oxide detoxification, had decreased content upon acetic acid - induced PCD (Table S1). Consistently, our data showed that acetic acid treatment results in decreased superoxide dismutase activity, particularly Sod1 (Fig. 4E). In contrast, the levels of a mitochondrial peroxiredoxin (Prx1) and a cytoplasmic peroxiredoxin (Ahp1), both involved in reduction of hydroperoxides, exhibited increased abundance in that same cell population (Table S1). It is possible that this dynamic alteration in the content of several proteins involved in the degradation of different ROS species, in particular of superoxide anion and hydrogen peroxide, may reflect the differential role that those species play in the PCD process in this strain, as previously proposed for *S. cerevisiae *27,28.

### Protein translation and folding

In addition to the alterations observed in the metabolic pathways described above, there was an increase in the abundance of two proteins involved in ribosome biogenesis (namely Rsm26 and one protein species of Rps5, proteins of the subunit of the mitochondrial and cytosolic small ribosomal subunits, respectively), one protein involved in protein translational quality control (the subunit of cytoplasmic phenylalanyl-tRNA synthetase Frs1), and one protein involved in translation elongation (Eft1), while two proteins involved in translation elongation (Tef2) and translational rate control (Ola1) exhibited the opposite behavior (Table S1). Apparently, these results are similar to the ones observed in *S. cerevisiae* cells under acetic acid - induced PCD 13. Most probably this is related with the fact that a global inhibition of protein synthesis is a common response to stress conditions 29 and mainly unrelated with PCD or resistance to acetic acid.

Acetic acid challenged cells also showed altered levels of several chaperones. Interestingly, the levels of Hsp60 and Ssc1, two chaperones described to be involved in protein import into the mitochondria, Por1, an outer membrane protein previously described as being important for the entry of metabolites in the mitochondria and having a role in PCD triggered by acetic acid 30, and Mas1, involved in processing of mitochondrial imported proteins, showed decreased levels upon acetic acid - induced PCD (Table S1). However, other chaperones involved in protein folding and regulation of translation fidelity (Ssb1, Ssa1 and Hsp26) showed increased abundance in that same cell population (Table S1). Curiously, Ssb1 and Ssa2, members of the Hsp70 family were found decreased in* S. cerevisiae *cells challenged with acetic acid, which might contribute for the higher susceptibility of this species to acetic acid given their role on stress response 14. In addition, Hsp70 proteins are crucial for the AIF (apoptotic inducing factor) translocation from the nucleus and PCD, an event known to occur in *S. cerevisiae* 31. The content of Hsc82, which has been previously implicated in *S. cerevisiae *PCD by promoting yeast survival 32, also exhibited decreased levels under acetic acid - induced PCD in ISA1307, as indicated either by quantitative proteomic analysis (Table S1) or quantitative immunoblot analysis performed using anti-HSP90 antibody, which detects both protein species of the HSP90 chaperone family, Hsc82 and Hsp82 (Fig. 2B). Given the controversial role of the members of the Hsp90 in yeast PCD 32 caution should be taken on the conclusions made about these observations.

### Amino acid and nucleotide metabolism

A significant number of changes registered in the mitochondrial protein content in acetic acid challenged cells were found to be related with amino acid metabolism. In particular, 28 protein species, corresponding to 22 unique proteins, involved in amino acid metabolism exhibited altered abundance upon acetic acid treatment (Table S1). Of these, several proteins involved in biosynthesis of methionine and cysteine (Met6, Met3, Met17, Cys4), as well as in biosynthesis of tryptophan (Trp5, Aro4), showed increased abundance in the acetic acid challenged population (Table S1). On the contrary, the abundance of several proteins involved in branched-chain amino acid biosynthesis (Ilv1, Ilv3, Ilv5, Ilv6) was decreased (Table S1). Three proteins involved in glutamate metabolism also showed altered content upon acetic acid - induced PCD. Whereas the levels of Pro2 and Idp2, two proteins involved in degradation and biosynthesis of glutamate, respectively, exhibited increased amounts in the presence of acetic acid - treated cells, the levels of Put2, involved in glutamate catabolism, exhibited decreased levels (Table S1). Some proteins involved in serine, glycine, lysine and arginine metabolism were also found to have an altered content under acetic acid stress (Table S1). Altogether, these results suggest that acetic acid affects the dynamics of the intracellular amino acid pool, as already demonstrated for *S. cerevisiae *cells under acetic acid stress 13, as well as under stress induced by the weak acid herbicide 2,4-dichlorophenoxyacetic acid 33. However, the profile of amino acid alterations found in both yeast strains denote different pathways involved and deserve further investigation.

Beside the proteins involved in amino acid metabolism, numerous proteins involved in nucleotide biosynthesis (Ade1, Ade12, Ade17), namely purine nucleotide biosynthesis, were found to have a higher abundance level in response to acetic acid - induced cell death (Table S1). In addition, a protein involved in spermidine biosynthesis (Spe3) was found to have increased content under acetic acid - induced PCD. Since spermidine has been reported to be involved both in the induction of cell death 34 and in the protection 35,36,37,38 from cell death depending on the cellular physiological conditions, and the mitochondrial proteome of ISA1307 cells, but not *S. cerevisiae *(Table S1 and our unpublished results), revealed an increase of Spe3 abundance in acetic acid treated cells, this led us to investigate the role that spermidine might play in ISA1307 higher tolerance to acetic acid.

### Spermidine protects ISA1307 and *S. cerevisiae* from acetic acid - induced PCD

**Figure 5:**
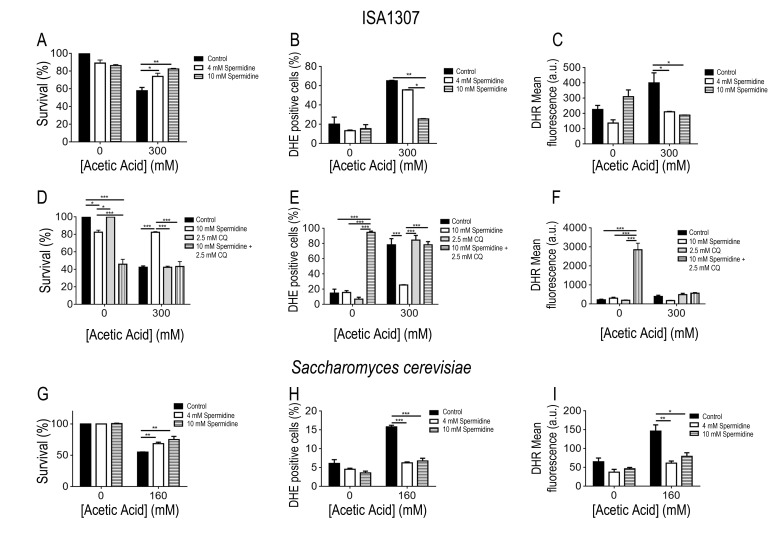
FIGURE 5: Spermidine protects cells from acetic acid - induced PCD. Comparison of the survival rate of **(A)** ISA1307 and **(G)** *Saccharomyces cerevisiae *cells treated with the described concentration of acetic acid combined with 4 mM or 10 mM spermidine. FACS measurements of superoxide anions (**B** and **H**) using the probe dihydrioethidium and hydrogen peroxide (**C** and **I**) using the probe dihydrorhodamine 123 (DHR) in ISA1307 and *S. cerevisiae* cells, respectively. **(D) **Survival rate and FACS measurements of **(E)** superoxide anions and **(F) **hydrogen peroxide (DHR) of ISA1307 cells treated with 300 mM of acetic acid combined with 10 mM spermidine and the autophagy inhibitor chloroquine (CQ) at 2.5 mM. Bar graphs indicate mean and standard error of the mean (SEM). Fluorescence/cell (arbitrary units) measured in 25,000 cells/sample in three independent experiments. Significance of the data was determined by two-way ANOVA (*P < 0.05; **P < 0.01; ***P < 0.001).

Extracellular administration of 4 mM spermidine to ISA1307 cells resulted in increased resistance and protection against acetic acid treatment (Fig. 5A), accompanied by reduced oxidative stress revealed by decreased accumulation levels of superoxide anion (Fig. 5B) and hydrogen peroxide (Fig. 5C), measured using dihydroethidium (DHE) and dihydrorhodamine 123 (DHR), respectively 39. Furthermore, the protective effects mediated by spermidine were concentration dependent, since when cells were subjected to 10 mM of spermidine a higher resistance to acetic acid was observed (Fig. 5A), accompanied with a drastic reduction of the superoxide anion levels (Fig. 5B).

In *S. cerevisiae*, spermidine administration also protected against acetic acid - induced cell death as observed in ISA1307 (Fig. 5E). In fact, the administration of 4 mM spermidine is sufficient to confer protection to *S. cerevisiae* cells against acetic acid (Fig. 5E-G), and no major differences were observed when compared to cells treated with 10 mM spermidine (Fig. 5E-G). The increased resistance to acetic acid appears to be also correlated with a decrease in the production of superoxide anion upon acetic acid treatment (Fig. 5E-G). The effects of spermidine were found to be autophagy dependent as previously shown in *S. cerevisiae*. In fact, our data shows that inhibition of autophagy by chloroquine abrogates the pro-survival effects and decreased ROS accumulation promoted by spermidine supplementation of ISA1307 cells upon acetic acid - induced PCD (Fig 5D-F).

## DISCUSSION 

This study was designed to get insights into the mechanisms involved in acetic acid - induced PCD in the *Z. bailii*-derived hybrid strain ISA1307 by analyzing the yeast mitochondrial protein expression profile of cells challenged by acetic acid. In the context of the present study, this interspecies hybrid strain offers the possibility to quantitatively assess allele - specific gene product expression. So far, few studies have already examined protein expression in hybrid strains, particularly in several industrial lager-brewing yeasts. The goal of some of these studies was to identify the parental strains or define relatedness and investigate taxonomic relationships between strains 40,41,42. Other analyses were focused on the specific quantitative differences registered in the proteome of those hybrid strains, during production - scale fermentation 42, standardized laboratory growth conditions 43 and under various stress conditions 44. In our study, we found that about 55% of the ISA1307 proteins with altered abundance during acetic acid - induced PCD were apparently derived from the parental *Z. bailii* species (99 - 100% nucleotide identity of the protein - encoding ORF with CLIB213^T^ genome), with the remaining proteins (about 45%) being derived from the other yeast species closely related to *Z. bailii* (94 - 98% nucleotide identity of the protein - encoding ORF with CLIB213^T^ genome) (Table S2). Overall, the majority of the proteins identified showed a coordinated regulation in both the *Z. bailii*-like protein and its non-*Z. bailii* counterpart and it is not possible to attribute a major contribution to the PCD process to either one or the other parental species. Interestingly, however, there were a few exceptions, namely for the *Z. bailii*-like species of the proteins 3-phosphoglycerate kinase Pgk1 and the mitochondrial alcohol dehydrogenase Adh3, involved in glycolysis/gluconeogenesis and fermentation, respectively, which were more abundant in the acetic acid challenged cell population, while their non-*Z. bailii* counterparts were less abundant in that same cell population (Table S1 and Table S2). It remains to be seen if these differences have some physiological relevance.

Remarkably, in this study, it was possible to identify by MS about 88% of the proteins whose content was found to be altered in the 2-DE gels, which contrasts with what had been found in a previous proteomic experiment in which only 40% of the protein spots considered of interest had been identified 13. The higher percentage of proteins identified in the analysis carried out in this work might reflect the higher conservation of the mitochondrial pathways involved in PCD in different yeast species. However, it also highlights the advantages of having available the annotated genome sequence of the organism under study 18.

The most representative functional classes found to be important for PCD in ISA1307 were metabolism and energy, indicating the central role of cellular metabolic processes in the regulation of PCD. Most of the changes observed upon acetic acid - induced PCD involved a generalized decrease in the protein content of enzymes of the tricarboxylic acid (TCA) cycle pathway, mitochondrial energetics and the pyruvate dehydrogenase (PDH) bypass. In addition, increased abundance of proteins involved in fermentation was also observed. Several proteins known to be involved in glycolysis/gluconeogenesis had also altered content in acetic acid challenged cells. Interestingly, we observed the existence of a few glycolytic/neoglucogenic proteolytic fragments (Eno1 and Cdc19) in the cell population under acetic acid treatment, which has been previously reported to occur during proteome changes upon a switch from respiratory to fermentative metabolism 42,45. In *S. cerevisiae*, during acetic acid - induced PCD, stimulation of the PPP was found to occur concomitantly with a decrease in glycolysis. This behavior was attributed to a decrease in Tpi1 abundance in acetic acid - challenged cells and it seems to be part of the acetic acid - PCD pathway in this yeast species 14. In our analysis, however, we observed no such generalized decrease in the content of glycolytic enzymes, namely in the content of Tpi1, which was even increased in the acetic acid - challenged cell population. This suggests that this might be a crucial difference between the metabolic re-programming happening during acetic acid - PCD in these two yeast species. Indeed, an yeast strain lacking *YCA1*, the yeast metacaspase, shows lower levels of certain PPP intermediates when compared with a WT strain and, although it also commits to an acetic acid - induced PCD program, it shows a lower death rate than the WT 14, suggesting that these differences might be responsible for differential cell survival in these conditions, which might also be the case for the ISA1307 strain.

A remarkable number of changes in protein content registered in this work is related to amino acid metabolism. Our results suggest increased biosynthesis of certain amino acids in the acetic acid challenged cell population, as reported before for acetic acid - induced PCD cells of *S. cerevisiae *found to be starved for several amino acids 13. Nevertheless, in ISA1307 cells undergoing acetic acid - induced PCD a particular regulation of glutamate metabolism, not seen in *S. cerevisiae*, seems to play a crucial role upon acetic acid treatment. A negative correlation between the intracellular level of glutamate and the level of propionic acid supplementation was reported and tentatively explained as a mechanism to maintain cytosol electrical neutrality by exchange of glutamate and the counterion propionate under propionic acid stress 46, a mechanism already reported to occur in acetic acid stressed *Escherichia coli *47 and suggested to take place in ISA1307 cells growing in the exponential phase in the presence of acetic acid^17^,^48^. Additionally, glutamate is a precursor for the synthesis of glutathione, which is a compound with important antioxidant properties known to play a role in PCD 49,50. In *S. cerevisiae,* depletion of glutathione induces PCD, a process, which is possibly related to ROS signaling – induced PCD 51. The results obtained in this proteomic analysis are also suggestive of the role played by the one-carbon folate metabolism, an essential process for the synthesis of purines and the regeneration of methionine in yeast, upon acetic acid induced PCD 52. This role is suggested by the increased content of the serine hydroxymethyltransferase Shm2, responsible for reversible folate-dependent conversion of serine to glycine, as well as by the increased abundance of methionine and purine biosynthetic enzymes in acetic acid challenged cells. In addition, the S-adenosylmethionine synthetase Sam2 was found to have decreased abundance in acetic acid challenged cells. Limited methionine availability in the cell was reported to be involved in chronological yeast aging, conferring protection against PCD by inhibiting cellular ROS over-accumulation 53.

In contrast, several proteins involved in branched-chain amino acid biosynthesis exhibited decreased content upon acetic acid induced PCD, suggesting a decreased activity of this biosynthetic pathway. Strikingly, aromatic amino acids (including tryptophan), methionine and branched amino acid are known to be involved in the Ehrlich pathway, which has been implicated in these amino acids catabolism. In fact, several proteins belonging to this pathway (Aro8, Bat1, Pdc1, Ald4 and Adh3) exhibited increased content in acetic acid challenged cells. Interestingly, an unusually high number of pyruvate decarboxylases genes was predicted to exist in ISA1307 genome, which was proposed to represent a specific niche adaptation of this strain to the considerable amounts of those amino acids that are present in wine 18. Since the first step of the Ehrlich pathway involves conversion of α-ketoglutarate into glutamate, it is possible that the activation of this pathway could represent another cellular strategy to replenish this amino acid. Glutamate balance seems to be important under acetic acid stress, which is also consistent with reduced branched chain amino acid synthesis since valine and isoleucine biosynthesis requires deamination of L-glutamate into α-ketoglutarate 54. In addition, fusel alcohols or acids, the end-products of the Ehrlich pathway, have been previously described as putative quorum-signaling molecules, playing a role in yeast adaptation to environmental challenges 55. For instance, in *S. cerevisiae*, the presence of 2-indole acetate induces pseudohyphal form, a process mediated by the oxidative stress responsive transcription factor Yap1 56. Formation of pseudomycelium-like structures in an ISA1307 population challenged with sub-lethal concentrations of acetic acid has been observed (our unpublished results).

In *S. cerevisiae*, the protein synthesis machinery is compromised during acetic acid - induced PCD 13,32 and the restoration of the translation machinery increases cell survival during exposure to PCD - inducing concentrations of the acid 32. That also seems to be the case in our analysis, since the content of both Tef2 and Ola1 were decreased upon acetic acid - induced PCD. However, since the content of several protein species involved in ribosome biogenesis was found to be increased upon acetic acid – induced PCD, and considering that it is becoming increasingly recognized that general translation is inhibited to allow the translational control of specific mRNAs required for survival during growth under stress conditions 32, it is possible that selective translation taking place in ISA1307 might underlie some of the differences on acetic acid resistance between both species. A general decline in the abundance of chaperone proteins involved in mitochondrial protein translocation and folding (Ssc1, Hsp60 and Hsc82) was also registered, which is consistent with the reduced mitochondrial activity predicted to occur in acetic acid treated cells. Differently, the amount of cytoplasmic chaperones (Ssb1, Ssa1 and Hsp26) increased, allowing a cytoplasmic chaperone – assisted folding, which could be important for selective translation of the several proteins found to have increased content in acetic acid challenged cells. This is also in clear contrast to what was previously observed in *S. cerevisiae*, in which a general decrease in several heat shock proteins was observed upon acetic acid – induced apoptosis, supposedly to allow a decrease in anti-apoptotic activity in the cell and the progression of acetic acid – induced PCD 14. It is thus possible that the increased amount of some cytoplasmic chaperones can explain, in part, ISA1307 increased tolerance to acetic acid – induced PCD.

The acetic acid treatment tested in this study was also found to lead to changes in the abundance of several proteins involved in cellular oxidative stress response upon acetic acid exposure (Table S1). In *S. cerevisiae*, it is well established that ROS, namely hydrogen peroxide, is a mediator of the PCD induced by acetic acid, being produced shortly upon acetic acid exposure, and leading to cytochrome *c *release and caspase activation in a ROS-dependent manner 27,57. This process is likely to involve the modulation of the cell antioxidant system as overexpression of cytosolic catalase confers protection against acetic acid - induced PCD 28.

The increased abundance of two peroxiredoxins detected in acetic acid challenged cells, together with the concomitant decrease of superoxide dismutase levels and enzymatic activity, suggests that dynamic modulation of ROS occurs during acetic acid - induced PCD in strain ISA1307. Interestingly, the abundance of the cytoplasmic thioredoxin peroxidase Tsa1 decreased upon acetic acid - induced PCD, in contrast to what was seen for the other peroxiredoxins identified in this study. While it has been generally considered that all peroxiredoxins share similar antioxidant functions, although having different expression and cellular localization 58, Tsa1 has been recently demonstrated to be unique among the other peroxiredoxins by acting in concert with cellular DNA repair processes to confer protection against lethal levels of DNA damage in yeast, which might occur during aerobic growth and potentially affect cell survival 59. It is thus likely that the differential expression of this protein reflects a distinct role played during acetic acid – induced PCD. In addition, the abundance of the nitric oxide synthase Yhb1 was also decreased in acetic acid challenged cells, which is consistent with the fact that lethal concentrations of acetic acid do not induce nitrosative stress in *S. cerevisiae *60.

Our results also revealed that spermidine supplementation protects both ISA1307 and *S. cerevisiae* cells from acetic acid - induced apoptosis, a process that involved decreased accumulation of oxidative stress species, namely superoxide anion and hydrogen peroxide, and is dependent on autophagy. However, while in *S. cerevisiae* cells the extracellular administration of spermidine confers protection against acetic acid, no alterations in Spe3 mitochondrial levels were observed upon acetic acid treatment, contrary to what was observed for ISA1307 (unpublished results). These results suggest that spermidine synthesis might not be increased, and therefore not protecting *S. cerevisiae* mitochondria from superoxide anion production, making those cells more susceptible to acetic acid treatment than ISA1307 cells and being related to the high acetic acid tolerance profile presented by this yeast strain.

Overall, the analysis of the mitochondrial - enriched proteome of a cell population under acetic acid - induced PCD, compared with the same unstressed population, evidenced a marked down-regulation of most mitochondrial functions, while the abundance of the majority of the non-mitochondrial proteins (about 80%) increased. This response suggests that PCD is well underway in the majority of the cell population and the mitochondria vital functions are shut down while mitochondrial death functions are increased. Alternatively, a large representation in the acetic acid challenged population of cells that are responding to the stress and thus reducing the production of ROS, could also be considered 61. In *Z. bailii,* respiration is not repressed by glucose as it is the case in *S. cerevisiae* where even in the presence of oxygen respiration is repressed, leading to reduced oxidative stress and inhibition of PCD 62. Possibly due to the negligible respiratory capacity of the *S. cerevisiae* strains tested under glucose repression, the mitochondrial ultrastructural alterations observed under acetic acid - induced PCD in *Z. bailii *were not detected in *S. cerevisiae *7. This fact suggests that *Z. bailii *may be used as a novel yeast model to study the evolutionary origins of the role of mitochondria in PCD pathways.that the metabolic changes observed might not be due to a non-specific, broad effect resulting from mitochondrial dysfunction, but instead consist in a specific cellular response to the acetic acid - induced stress, further reinforcing the emerging concept that metabolic processes, especially those linked to mitochondrial signaling events, are crucial regulators of the type of PCD that is activated in the cell, namely apoptosis or programmed necrosis recently described (necroptosis) 63,64.

## Materials and Methods

### Strains and growth conditions

The *Zygosaccharomyces bailii *- derived interspecies hybrid strain ISA1307 was batch-cultured at 30°C with orbital agitation (150 rpm) in mineral medium (MK) 65 with vitamins, oligo-elements and 2% (w/v) glucose. For *Saccharomyces cerevisiae* BY4741 cultivation, MK medium was supplemented with 20 mg methionine, 20 mg histidine, 60 mg leucine, and 20 mg uracil. In all experiments, MK pH was adjusted to 3.0. Yeast Peptone Dextrose (YPD) growth medium, containing, per liter, 20 g glucose, 20 g bactopeptone and 10 g yeast extract, was used for the assessment of viable cells’ concentration. Solid YPD growth media was obtained by supplementing the corresponding liquid medium with 2% agar.

### Acetic acid susceptibility assays

The susceptibility of ISA1307 cells to acetic acid was assessed by comparing the concentration of viable cells in liquid MK medium (at pH 3.0) either or not supplemented with acetic acid concentrations inducing approximately a 50% decrease in cell viability. For acetic acid treatment, yeast cells were grown until the early stationary phase (OD_640nm _= 3.6) in liquid MK medium. Cells were harvested and suspended (OD_640nm _= 1.0) in fresh medium (pH 3.0) followed by the addition of 300 mM acetic acid and incubation during 200 min at 30°C with stirring (150 rpm). After treatment, approximately 300 cells were spread on YPD agar plates and viability was determined by counting colony-forming units after 2 days of incubation at 30°C. For proteomic analysis, experiments were performed in MK medium and an equitoxic dose of acetic acid, inducing 50% of cell death shown to be of apoptotic nature as evaluated by terminal deoxynucleotidyl transferase - mediated dUTP nick end labeling (TUNEL) assay after 200 min.

### Sampling, protein fractionation and separation by two-dimensional electrophoresis (2-DE)

#### Sampling and protein extraction

For proteomic analysis, mitochondria were isolated according to the protocol previously described 21 with minor adaptations. Briefly, 2 L of cells were harvested at 4000 x *g*, 4°C, during 30 min and washed once with 2 M sorbitol. Cell wall was digested with zymolyase buffer (2 M sorbitol-D, 1 M phosphate buffer (pH 7.5), Zymolyase 20.000 U, 125 mM β-mercaptoethanol, and 0.5 M EDTA) at 30°C for 1 hour. Spheroplasts were washed thrice with 1.2 M sorbitol and were disrupted with lysis buffer (0.5 M sorbitol-D, 20 mM tris, 1 mM EDTA, and 2.85 mM phenylmethanesulphonyl fluoride (PMSF)) using a Potter homogenizator. Mitochondria extracts were separated, washed by high-speed centrifugation at 10000 x *g *for 15 min at 4°C (Beckman Coulter, JA-25.50 Rotor) and resuspended in sorbitol buffer (0.5 M sorbitol-D, 5 mM EDTA, and 50 mM tris). Protein concentration of the extracts obtained was determined using the Pierce BCA protein assay kit (Thermo Scientific). For proteomic analysis, total mitochondrial protein (90 μg for analytical gels or 400 μg for preparative gels) was precipitated with the 2D-CleanUp kit (GE Healthcare) and resuspended in 80 µL of rehydration solution (8 M urea, 4% w/v CHAPS, 0.5% v/v Pharmalytes 3-10, 15 mM DTT and traces of bromophenol blue).

#### Protein separation by 2-DE

2-DE was performed as previously described 17. In order to reduce technical variation that may occur across different separations, four 2-DE gels for each experimental condition were analyzed (technical replicates). To reduce biological variation, each biological sample was prepared by pooling together cell samples obtained from three independent growth experiments (biological replicates). After 2-DE, analytical gels were fixed and stained using Flamingo fluorescent gel staining, according to the manufacturer’s instructions (Bio-Rad). For preparative gels, gels were fixed and stained using Hot Coomassie and silver staining. The gels were scanned in a Typhoon Trio scanner (GE Healthcare) and the gel images were analyzed using Progenesis SameSpots (Nonlinear Dynamics) software as previously described 17.

### *in-gel* digestion and MALDI-TOF/TOF analysis

Protein spots excised from 2-DE gels were used for *in gel* digestion. Briefly, the protein spots were reduced and alkylated with dithiothreitol (DTT, 10 mM in 100 mM ammonium bicarbonate) and iodoacetamide (IAA, 55 mM in 100 mM ammonium bicarbonate), respectively. After this the gel plugs were dried and incubated with modified trypsin (6.7 ng/μL in 50 mM ammonium bicarbonate) at 37°C overnight. The obtained peptide supernatants were desalted and concentrated with chromatographic microcolumns using GELoader tips packed with POROS R2 (Applied Biosystems, 20 µm bead size) and then directly eluted onto the MALDI target plate using 0.6 µl of 5 mg/ml α-CHCA (α-ciano-4-hydroxy-cinnamic acid) in 50% (v/v) acetonitrile with 5% (v/v) formic acid and air-dried.

Tandem mass spectrometry analysis was performed using a MALDI-TOF/TOF 4800 *plus* mass spectrometer (Applied Biosystems). The equipment was calibrated using angiotensin II (1046.542 Da), angiotensin I (1296.685 Da), neurotensin (1672.918 Da), ACTH (1-17) (2093.087 Da), and ACTH (18-39) (2465.199) (Peptide Calibration Mixture 1, LaserBio Labs). Each reflector MS spectrum was collected in a result - independent acquisition mode, typically using 750 laser shots per spectra and a fixed laser intensity of 3200V. The fifteen strongest precursors were selected for MS/MS, the strongest precursors being fragmented first. MS/MS analyses were performed using CID (Collision Induced Dissociation) assisted with air, with collision energy and gas pressure of 1 kV and 1 x 10^6^ torr, respectively. Each MS/MS spectrum collected consisted of 1400 laser shots using a fixed laser intensity of 4300V.

### Protein identification

Protein identification was performed using ProteinPilot software (version 4.5, AB Sciex, Framingham, MA) coupled to MASCOT (version 2.2, Matrix Science, Boston, MA) search engine. Searches were performed using combined analysis of the intact masses of the tryptic peptides (MS) and tandem mass data (MS/MS). Search parameters were set as follows: minimum mass accuracy of 50 ppm for parent ion, an error tolerance of 0.3 Da for fragments, maximum two missed cleavage in peptide masses, and carbamidomethylation (C), oxidation (M), deamidation (NQ), Gln -> pyro-Glu (N-term Q) were set as variable amino acid modifications and the ISA1307 strain database (2013) (9927 sequences; 4855012 residues) was used 18. Peptides were only considered if the ion score indicated extensive homology (P < 0.05). Proteins were considered if having significant MASCOT score (minimum score 52) and at least one peptide with extensive sequence homology.

### Western blot analysis

For detection of protein levels by SDS-PAGE and Western blot in total cellular extracts, untreated or acetic acid – treated cells (300 mM) were collected and disrupted using glass beads in lysis buffer (1% v/v Triton X-100, 120 mM NaCl, 50 mM Tris-HCl pH 7.4, 2 mM EDTA, 10% v/v Glycerol, 1 mM PMSF and Complete Mini protease inhibitor cocktail (Roche, Mannheim, Germany)). Of total protein, 20 μg were resolved on a 12% SDS gel and transferred to a nitrocellulose membrane (Bio-Rad, 170-4159) during 7 min at 25V. The membranes were blocked with tris buffered saline (TBS) with 0.1% Tween 20 (TBST) containing 5% skim milk, followed by incubation with polyclonal rabbit anti-Tef1/2 (1:15000, kindly supplied by Prof. Kinzy, T.G.), monoclonal rat anti-HSP90 (1:1000, Calbiochem), polyclonal rabbit anti-Eft1/2 (1:1000), polyclonal goat anti-Aco1/2 (1:1000), polyclonal mouse anti-Por1 (1:1000, Invitrogen), and anti-actin (1:5000) (kindly provide by Dr. Gourlay, C.) primary antibodies. After washing with TBS, the membranes were incubated with the respective secondary antibody at a dilution of 1:5000 and detected by enhanced chemiluminescence.

### Superoxide dismutase assays

For determination of superoxide dismutase activities, yeast extracts were prepared in 25 mM Tris buffer (pH 7.4) containing a cocktail of protease inhibitors. Protein content of cellular extracts was estimated by the method of Bradford (Bio-Rad) using bovine serum albumin (BSA) as a standard. Superoxide dismutase activities were measured based on their ability to inhibit reduction of nitro blue tetrazolium (Sigma, N6876) to formazan in non-denaturing polyacrylamide gels 66.

### TUNEL assay

DNA strand breaks were assessed by a terminal deoxynucleotidyl transferase-mediated dUTP nick end-labeling (TUNEL) assay with the In situ Cell Death Detection Kit, Fluorescein (Roche Applied Science, Indianapolis, IN, U.S.A.). Yeast cells were initially fixed with 3.7% formaldehyde followed by digestion of the cell walls with lyticase. After preparation of cytospins, the slides were rinsed with PBS, incubated in permeabilization solution (0.1%, v/v, Triton X-100 and 0.1%, w/v, sodium citrate) for 3 min on ice, rinsed twice with PBS, and incubated with 10 µl of TUNEL reaction mixture (terminal deoxynucleotidyl transferase and FITC-dUTP) for 60 min at 37°C 6. Finally the slides were rinsed three times with PBS and a coverslip was mounted with a drop of anti-fading agent Vectashield (Molecular Probes, Eugene, OR, U.S.A.) and with 2 µl of 50 µg/ml propidium iodide (PI, Molecular Probes, Eugene, OR, U.S.A.) solution in Tris buffer (10 mM, pH 7.0) with MgCl_2_ (5 mM) and RNase (0.5 µg/ml). Cells were visualized with an Olympus PlanApo 60X/oil objective, with a numerical aperture of 1.42. For quantification of the number TUNEL positive cells, at least 400 cells from three independent assays were counted. Data express the percentage of TUNEL positive cells compared to the total number of counted cells.

### Respiration assay

At the appropriate time, a volume equivalent to* ~*2 OD_640_ units of ISA1307 cells treated with 300 mM of acetic acid for 200 min or untreated cells was harvested*. *Cells were resuspended in a solution containing 0.4 ml of a 0.4% (w/v) aqueous solution of 2-(p-iodophenyl)-3-(p-nitrophenyl)-5-phenyl tetrazolium chloride (INT; Sigma) and 0.1 ml of culture medium with or without acetic acid to a final concentration of 300 mM (in the 0.5 ml) to maintain the selective pressure. Cells were then incubated at 30°C for 30 minutes. After incubation, cells were harvested by centrifugation and the supernatants were discarded. One ml of undiluted DMSO was added to the cell pellet and the sample was sonicated for 15 s to favour cell disruption and solubilisation of formazan crystals. Cells were pelleted by centrifugation and the final absorbance of DMSO extracts was measured at 480 nm.

### Assessment of intracellular superoxide anion accumulation

Free intracellular ROS, specifically superoxide anions were measured using dihydroethidium (DHE) and hydrogen peroxide with dihydrorhodamine 123 (DHR) as previously described 67. Briefly, aliquots of cells were collected and DHE was added to a final concentration of 5 µM from a 5 mM stock in DMSO and cells were incubated for 10 min at 30°C. For DHR staining, aliquots were taken and DHR was added to a final concentration of 15 µg/mL and cells were incubated for 90 min at 26°C. After incubation, cells were washed once with PBS. The DHE signals were measured using FACSCaliber2 flow cytometer (BD-Biosciences) with a 488 nm excitation laser. The DHR signal was collected through a 488-nm blocking filter, a 550-nm long-pass dichroic with a 525 nm band pass. Signals from 30,000 cells/sample were captured at a flow rate of 1,000 cells/s. Data collected with the FACSCaliber2 flow cytometer were processed with Flowjo software (Tree Star) and quantified with WinList software (Verity Software House).

### Spermidine supplementation and chloroquine assays

For treatment with spermidine or spermidine plus chloroquine (CQ), yeast cells were grown until the early stationary phase (OD_640nm _= 3.6) in liquid MK medium. Cells were harvested and suspended (OD_640nm _= 1.0) in fresh medium (pH 3.0) followed by the addition of 300 mM acetic acid and 4 mM or 10 mM of spermidine or 2.5 mM CQ plus 10 mM spermidine. Cells were incubated during 200 min at 30°C with stirring (150 rpm). After treatment, approximately 300 cells were spread on YPD agar plates and viability was determined by counting colony - forming units after 2 days of incubation at 30°C.

### Statistical analysis

For proteomic analysis, the built-in statistical analysis tools of Progenesis Samespots software were used. Averages for each growth condition were first compared by their normalized volume using one-way ANOVA between group test, and only those spots considered to be statistically significant (p-value below 0.05) were selected for further analysis. To address the multiple hypothesis testing problem, adjusted P-values (q-values) were then calculated using an automatic optimized FDR approach, and only spots with a q-value below 0.05 were included in our analysis. Statistical analysis for other experiments was carried out using Graph Pad prism 5 software. Significance of the data was determined by two-way ANOVA. The threshold for statistical significance was set to P = 0.05.

## SUPPLEMENTAL MATERIAL

Click here for supplemental data file.

All supplemental data for this article are also available online at http://microbialcell.com/researcharticles/mitochondrial-proteomics-of-the-acetic-acid-induced-programmed-cell-death-response-in-a-highly-tolerant-zygosaccharomyces-bailii-derived-hybrid-strain/.
