# Supplementary material for: Mitochondrial proteomics of the acetic acid - induced programmed cell death response in a highly tolerant Zygosaccharomyces bailii - derived hybrid strain
Source: Microb Cell. 2016 Jan 22;3(2):65–78. doi: 10.15698/mic2016.02.477 (PMC5349105; doi:10.15698/mic2016.02.477)
Supplement: Supplementary file 1 [file mic-03-065-s01.pdf]

Figure S1. Graphical representation of the interaction network of ISA1307 proteins shown to have an altered content during acetic acid-induced PCD. The protein interaction network map shown here was adapted from the output of STRING database [1], using the default parameters except for the active prediction methods that were set to experiments and databases only. Modes of action are shown in different colors: binding (blue lines), reaction (black connectors), catalysis (brown lines) and activation (green arrows). Thicker, light brown lines indicate protein association described in curated databases. As ISA1307 genome is not available in the STRING database yet, this analysis was made using *S. cerevisiae* homologs of the identified proteins. The main functional groups identified in this analysis as being relevant to acetic acid-induced PCD are highlighted.

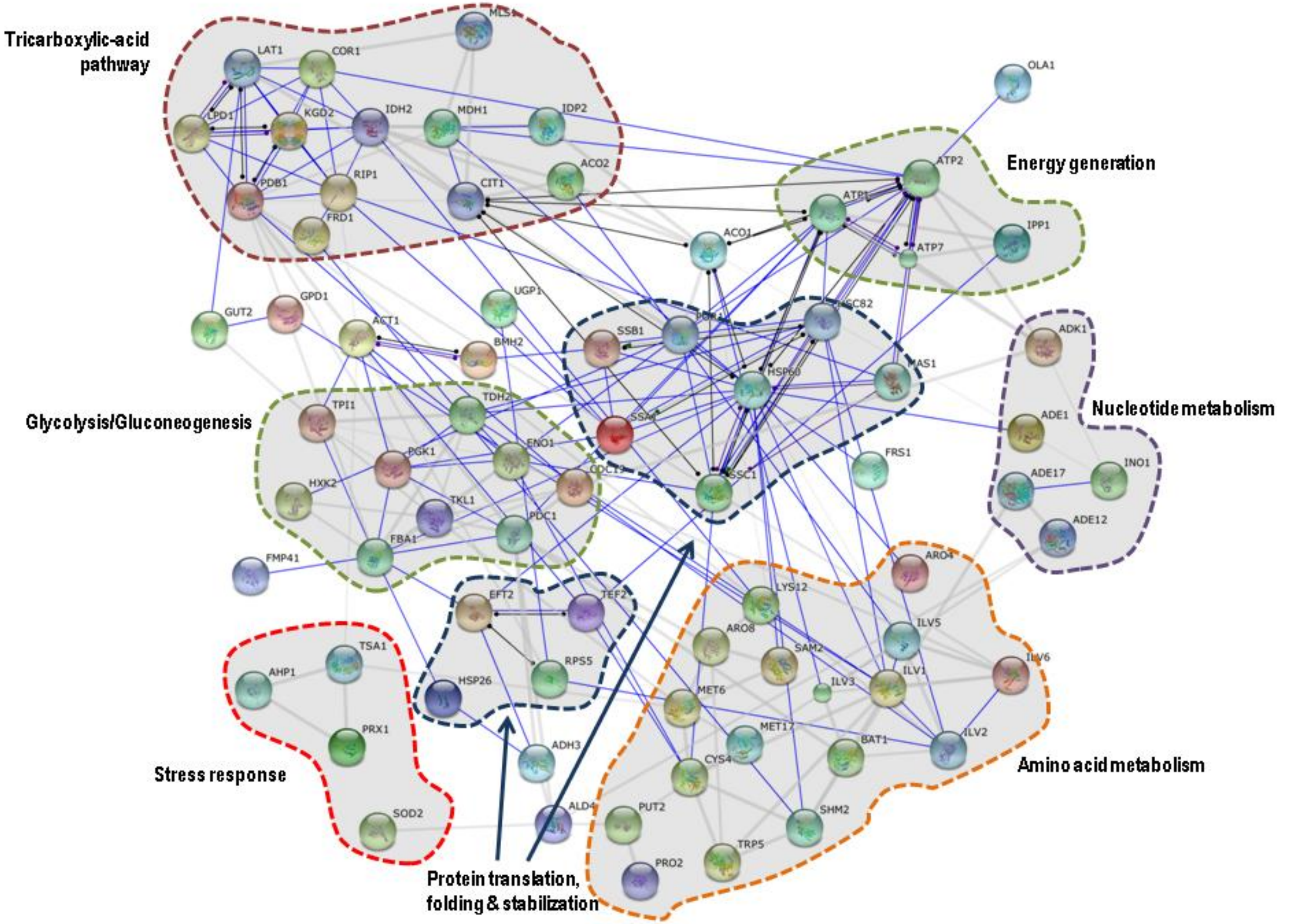
Tricarboxylic-acid pathway
Energy generation
Glycolysis/Gluconeogenesis
Nucleotide metabolism
Stress response
Amino acid metabolism
Protein translation, folding & stabilization
LAT1
COR1
MLS1
LPD1
KGD2
IDH2
MDH1
IDP2
ACO2
PDB1
RIP1
CIT1
FRD1
OLA1
ATP2
ATP1
IPP1
ACO1
ATP7
GUT2
GPD1
ACT1
UGP1
BMH2
SSB1
SSC82
HSP60
MAS1
ADK1
ADE1
INO1
ADE17
ADE12
TPI1
TDH2
SSA1
PGK1
ENO1
CDC19
HXK2
TKL1
PDC1
FBA1
FMP41
FRS1
SSC1
EFT2
TEF2
RPS5
HSP26
AHP1
TSA1
PRX1
SOD2
ADH3
ALD4
ARO4
LYS12
ARO8
ILV5
SAM2
ILV6
ILV3
ILV1
MET6
MET17
BAT1
ILV2
CYS4
PUT2
SHM2
TRP5
PRO2

Table S1. Proteins with altered content (above 1.5-fold or below 0.7-fold) in strain ISA1307 under acetic acid-induced PCD.

[a]The putative function of each ORF was assigned based on the function of each *S. cerevisiae* homologous gene (www.yeastgenome.org). [b] Values were calculated as the average data from, at least, three independent experiments and data were filtered to retain spots with an ANOVA-associated *p*-value and an adjusted p-value (q-value) below 0.05. Fold changes correspond to ratio values of normalized protein spots intensities, in 2-DE gels, obtained using cells of strain ISA1307 grown for 200 min. in the absence of acetic acid versus cells grown for 200 min. in medium supplemented with a PCD inducing concentration of acetic acid. [c] p-values and [d] adjusted p-values (q-values), determined based on an optimized FDR approach for multiple testing, equal or below 0.05 were considered to ensure high statistical confidence of differential expression (with two exceptions, which are underlined).

**Spot number**	**Proteins identified**	***S. cerevisiae* homologs**	**Function[a]**	**Fold Change[b]**	**ANOVA[c]**	**q-value[d]**	**Predicted localization**
		**Metabolism**					
		Amino acid metabolism					
**83**	ZBAI_ 01577	Sam2	S-adenosylmethionine synthetase	0.4	1.31E-03	7.36E-03	Unknown
**8**	ZBAI_08766	Met6	Cobalamin-independent methionine synthase	5.1	9.62E-06	9.06E-04	Cytoplasm, Plasma membrane
**24**	ZBAI_01179	Met3	ATP sulfurylase	3.6	1.10E-05	9.06E-04	Cytoplasm, Mitochondrion
**10**	ZBAI_ 01779	Met17	Methionine and cysteine synthase	4.7	2.35E-03	9.91E-03	Cytoplasm, Plasma membrane

**41**	ZBAI_01779			3.1	3.59E-05	1.96E-03	
**3**	ZBAI_05689	Shm2	Serine hydroxymethyltransferase	6.1	1.24E-04	2.99E-03	Cytoplasm, Plasma membrane
**21**	ZBAI_01422			3.8	1.01E-03	6.66E-03	
**58**	ZBAI_01422			2.8	1.88E-03	8.90E-03	
**139**	ZBAI_ 03097	Ilv1	Threonine deaminase	0.5	6.31E-04	5.95E-03	Mitochondrion
**113**	ZBAI_02009	Ilv2	Acetolactate synthase	2.1	1.69E-02	3.29E-02	Mitochondrion
**97**	ZBAI_09663	Ilv3	Dihydroxyacid dehydratase	0.5	6.61E-04	5.95E-03	Mitochondrion
**7**	ZBAI_01751	Ilv5	Bifunctional acetohydroxyacid reductoisomerase	0.2	4.58E-04	5.03E-03	Mitochondrion
**36**	ZBAI_01751			0.3	1.30E-03	7.38E-03	
**158**	ZBAI_03439	Ilv6	Regulatory subunit of acetolactate synthase	0.6	1.24E-02	2.89E-02	Mitochondrion
**87**	ZBAI_04053	Bat1	Mitochondrial branched-chain amino acid (BCAA) aminotransferase	2.3	1.04E-02	2.42E-02	Mitochondrion
**162**	ZBAI_04053			0.6	5.00E-04	5.50E-03	
**34**	ZBAI_06832	Pro2	Gamma-glutamyl phosphate reductase	3.3	1.93E-04	3.69E-03	Cytoplasm, Nucleus
**35**	ZBAI_08072	Put2	Delta-1-pyrroline-5-carboxylate dehydrogenase	0.3	3.37E-04	4.64E-03	Mitochondrion
**72**	ZBAI_01085	Trp5	Tryptophan synthase	2.5	7.58E-04	6.21E-03	Cytoplasm, Nucleus
**81**	ZBAI_07777	Aro4	3-deoxy-D-arabino-heptulosonate-7-phosphate (DAHP) synthase	2.3	5.67E-04	5.60E-03	Cytoplasm, Nucleus
**82**	ZBAI_00799	Aro8	Aromatic aminotransferase I	2.3	3.91E-02	5.51E-02	Cytoplasm
**85**	ZBAI_00201	Cys4	Cystathionine beta-synthase	2.3	8.04E-03	2.23E-02	Cytoplasm, Mitochondrion
**157**	ZBAI_05297	Arg5,6	Protein that is processed in the mitochondrion to yield acetylglutamate	0.6	6.21E-03	1.76E-02	Mitochondrion
**159**	ZBAI_00128			0.6	9.87E-03	2.31E-02	

			kinase and N-acetyl-gamma-glutamyl-phosphate reductase				
**74**	ZBAI_06232	Lys9	Saccharopine dehydrogenase	2.4	7.29E-03	2.05E-02	Cytoplasm
**133**	ZBAI_00269	Lys12	Homo-isocitrate dehydrogenase	0.5	1.08E-03	6.66E-03	Mitochondrion
**63**	ZBAI_08441	Idp2	Cytosolic isocitrate dehydrogenase	2.6	2.29E-05	1.50E-03	Cytoplasm
**98**	ZBAI_00399	Spe3	Spermidine synthase	2.2	1.38E-02	3.23E-02	Cytoplasm, Nucleus
		Nucleotide/nucleoside/nucleobase metabolism					
**73**	ZBAI_08000	Ade1	N-succinyl-5-aminoimidazole-4-carboxamide ribotide (SAICAR) synthetase	2.4	3.61E-03	1.20E-02	Cytoplasm Nucleus
**121**	ZBAI_00026	Ade12	Adenylosuccinate synthase	2	1.64E-03	8.42E-03	Cytoplasm
**126**	ZBAI_00026	Ade1		2	2.83E-03	1.07E-02	Cytoplasm
**13**	ZBAI_06465	Ade17	Enzyme of 'de novo' purine biosynthesis	4.3	1.32E-03	7.51E-03	Cytoplasm, Plasma membrane
**14**	ZBAI_06465			4.2	1.85E-04	3.61E-03	
**44**	ZBAI_04908			3.1	5.77E-04	5.74E-03	
**135**	ZBAI_ 04908			1.9	3.34E-02	4.86E-02	
**140**	ZBAI_04908			0.6	1.77E-03	8.42E-03	
**137**	ZBAI_04734	Adk1	Adenylate kinase	0.3	2.66E-03	1.01E-02	Cytoplasm, Mitochondrion
		Phosphate metabolism					
**25**	ZBAI_07987	Ipp1	Cytoplasmic inorganic pyrophosphatase	3.6	7.25E-04	6.21E-03	Cytoplasm
**89**	ZBAI_07987			2.3	1.12E-03	6.72E-03	
		C-compound and carbohydrate metabolism					
**26**	ZBAI_00097	Ydl124w	NADPH-dependent alpha-keto amide	3.5	1.14E-03	6.88E-03	Cytoplasm, Nucleus

			reductase				
**51**	ZBAI_04106	Cat5	Protein required for ubiquinone biosynthesis	0.3	1.02E-03	6.66E-03	Mitochondrion
**15**	ZBAI_ 03268	Ino1	Inositol-3-phosphate synthase	4.2	1.10E-03	6.66E-03	Cytoplasm
**30**	ZBAI_01262	Bmh1	14-3-3 protein, minor isoform	3.4	1.05E-03	6.66E-03	Cytoplasm, Nucleus, Plasma membrane
**90**	ZBAI_02973	Gut2	Glycerol-3-phosphate dehydrogenase	2	3.13E-02	4.86E-02	Mitochondrion
**91**	ZBAI_02973			2.3	8.09E-04	6.31E-03	
**107**	ZBAI_02973			2.1	2.68E-03	1.46E-02	
**66**	ZBAI_ 03468	Ugp1	UDP-glucose pyrophosphorylase	2.6	7.35E-03	2.05E-02	Cytoplasm, Nucleus
		**Energy**					
		Glycolysis/ Gluconeogenesis					
**80**	ZBAI_08499	Fba1	Fructose 1,6-bisphosphate aldolase	2.3	8.98E-04	6.42E-03	Cytoplasm, Mitochondrion
**134**	ZBAI_03500			1.9	4.08E-03	1.33E-02	
**71**	ZBAI_04932	Cdc19	Pyruvate kinase	2.5	4.78E-05	2.01E-03	Cytoplasm, Plasma membrane
**123**	ZBAI_04932			0.5	7.17E-03	1.99E-02	
**4**	ZBAI_ 09271	Hxk2	Hexokinase isoenzyme 2	0.2	3.85E-06	8.20E-04	Cytoplasm, Mitochondrion, Nucleus
**6**	ZBAI_09271			0.2	3.41E-06	8.20E-04	
**55**	ZBAI_09271			0.4	5.74E-05	2.01E-03	
**155**	ZBAI_02835	Tpi1	Triose phosphate isomerase	1.7	5.94E-03	1.68E-02	Cytoplasm, Mitochondrion, Plasma membrane
**69**	ZBAI_01182	Tdh2	Glyceraldehyde-3-phosphate dehydrogenase	2.5	2.78E-05	1.58E-03	Cytoplasm, Mitochondrion
**70**	ZBAI_01182			2.5	2.48E-04	4.60E-03	
**31**	ZBAI_03405	Pgk1	3-phosphoglycerate kinase	3.4	4.66E-04	5.45E-03	Cytoplasm, Mitochondrion, Plasma membrane

**77**	ZBAI_08408			0.4	7.87E-04	6.31E-03	
**120**	ZBAI_03405			2	4.58E-03	1.51E-02	
**32**	ZBAI_04207	Eno1	Enolase I	0.3	7.14E-05	2.18E-03	Cytoplasm, Mitochondrion, Plasma membrane
**86**	ZBAI_07717			0.4	8.63E-03	2.23E-02	
**103**	ZBAI_07717			0.5	8.02E-04	6.42E-03	
**131**	ZBAI_07717			2	3.21E-02	4.86E-02	
		Glyoxylate cycle					
**19**	ZBAI_09166	Mls1	Malate synthase	4	6.62E-04	5.95E-03	Cytoplasm, Peroxisome
		Pentose phosphate pathway					
**45**	ZBAI_05562	Tkl1	Transketolase	3	5.58E-03	1.56E-02	Cytoplasm
**50**	ZBAI_05562			2.9	1.25E-03	7.13E-03	
		Pyruvate dehydrogenase complex					
**92**	ZBAI_05708	Lpd1	Dihydrolipoamide dehydrogenase	0.4	1.25E-03	7.13E-03	Mitochondrion
**96**	ZBAI_01441			0.5	5.61E-04	5.60E-03	
**84**	ZBAI_ 03035	Lat1	Dihydrolipoamide acetyltransferase component (E2) of pyruvate dehydrogenase complex	0.4	2.44E-03	1.01E-02	Mitochondrion
**114**	ZBAI_08717			0.5	1.25E-03	7.13E-03	
**163**	ZBAI_01905	Pdb1	E1 beta subunit of the pyruvate dehydrogenase complex	0.7	5.40E-03	1.56E-02	Mitochondrion
		Tricarboxylic-acid pathway (citrate cycle, Krebs cycle, TCA cycle)					
**12**	ZBAI_01777	Aco1	Aconitase	0.2	7.59E-04	6.21E-03	Mitochondrion
**60**	ZBAI_07063			0.4	1.99E-04	3.70E-03	

**115**	ZBAI_01777			0.5	1.80E-03	8.59E-03	
**148**	ZBAI_00463	Aco2	Aconitase	0.6	1.35E-02	3.11E-02	Mitochondrion
**154**	ZBAI_05617			0.6	2.58E-02	4.82E-02	
**150**	ZBAI_05994	Kgd2	Dihydrolipoyl transsuccinylase	0.6	4.64E-03	1.53E-02	Mitochondrion
**2**	ZBAI_04020	Mdh1	Mitochondrial malate dehydrogenase	0.2	6.74E-06	9.06E-04	Mitochondrion
**5**	ZBAI_04020			0.2	9.44E-06	9.06E-04	
**29**	ZBAI_04020			0.3	3.32E-04	4.61E-03	
**46**	ZBAI_04020			0.3	1.17E-02	2.76E-02	
**156**	ZBAI_03797	Idh2	Subunit of mitochondrial isocitrate dehydrogenase	0.6	3.32E-03	1.20E-02	Mitochondrion
**37**	ZBAI_08418	Cit1	Citrate synthase	0.3	1.28E-04	2.99E-03	Mitochondrion
**136**	ZBAI_03414			0.5	6.44E-04	5.95E-03	
		Respiration					
**18**	ZBAI_01038	Atp2	Mitochondrial ATP synthase, beta subunit	0.3	1.07E-03	6.66E-03	Mitochondrion
**64**	ZBAI_01038			0.4	3.08E-03	1.15E-02	
**116**	ZBAI_09839	Atp7	Subunit d of the stator stalk of mitochondrial F1F0 ATP synthase	0.5	1.82E-03	8.59E-03	Mitochondrion
**43**	ZBAI_03278	Cor1	Core subunit of the bc1 complex	0.3	5.04E-04	5.50E-03	Mitochondrion
**49**	ZBAI_08285			0.3	5.27E-04	5.50E-03	
**124**	ZBAI_08285			0.5	7.54E-04	6.21E-03	
**122**	ZBAI_08357	Rip1	Ubiquinol-cytochrome-c reductase (bc1 complex)	0.5	1.22E-02	2.80E-02	Mitochondrion
**125**	ZBAI_03351			0.5	3.87E-03	1.27E-02	

**127**	ZBAI_03351			0.5	5.53E-03	1.58E-02	
**143**	ZBAI_00484	Frd1	Fumarate reductase	1.8	2.69E-04	4.16E-03	Mitochondrion
**144**	ZBAI_00484			1.8	2.90E-03	1.10E-02	
**142**	ZBAI_06765	Ald4	Aldehyde dehydrogenase	1.8	3.62E-03	1.20E-02	Mitochondrion
**129**	ZBAI_08742	Por1	Mitochondrial porin	0.5	4.07E-03	1.36E-02	Cytoplasm, Mitochondrion, Plasma membrane
**132**	ZBAI_08742			0.5	1.46E-03	7.84E-03	
**146**	ZBAI_08742			0.6	1.83E-02	3.47E-02	
		Fermentation					
**17**	ZBAI_ 01038	Pdc1	Pyruvate decarboxylase isozyme	4.1	6.89E-05	2.14E-03	Cytoplasm, Nucleus
**52**	ZBAI_ 02995	Dld3	D-lactate dehydrogenase	2.9	1.85E-04	3.61E-03	Cytoplasm
**102**	ZBAI_02355	Adh3	Mitochondrial alcohol dehydrogenase isozyme III	2.2	1.15E-04	2.91E-03	Cytoplasm
**152**	ZBAI_02050	Adh3		0.6	5.13E-03	1.56E-02	Mitochondrion
		**Cell rescue, defense and virulence**					
		Stress response					
**54**	ZBAI_04388	Sod2	Mitochondrial superoxide dismutase	0.4	5.02E-05	2.01E-03	Mitochondrion
**100**	ZBAI_09380	Tsa1	Thioredoxin peroxidase	0.5	9.82E-04	6.67E-03	Cytoplasm
**166**	ZBAI_ 03696	Prx1	Mitochondrial peroxiredoxin	1.5	3.54E-02	5.07E-02	Mitochondrion
**20**	ZBAI_05673	Act1	Actin	3.9	1.43E-04	2.99E-03	Cytoplasm
**145**	ZBAI_05673			1.8	2.30E-03	9.67E-03	
**62**	ZBAI_08009	Gpd1	NAD-dependent glycerol-3-phosphate dehydrogenase	0.4	9.55E-03	2.24E-02	Cytoplasm, Nucleus, Peroxisome
**165**	ZBAI_09676	Yhb1	Nitric oxide oxidoreductase	0.7	8.27E-03	2.23E-02	Cytoplasm, Mitochondrion, Nucleus

**27**	ZBAI_ 02477	Ahp1	Thiol-specific peroxiredoxin	3.5	5.41E-05	2.01E-03	Cytoplasm, Plasma membrane
		**Protein synthesis and fate**					
		Ribosome biogenesis					
**42**	ZBAI_ 01036	Rps5	Protein component of the small (40S) ribosomal subunit	3.1	2.20E-05	1.49E-03	Cytoplasm
**68**	ZBAI_ 01036			0.4	6.20E-04	5.95E-03	
**9**	ZBAI_08296	Rsm26	Ribosomal protein of the small subunit	5.1	4.43E-05	1.99E-03	Mitochondrion
		Translation					
**48**	ZBAI_03803	Eft2	Translation elongation factor 2	2.9	1.60E-02	3.29E-02	Cytoplasm
**160**	ZBAI_ 03803			1.6	2.39E-02	4.46E-02	
**151**	ZBAI_ 00412	Tef2	Translational elongation factor 1 alpha	0.6	4.51E-03	1.47E-02	Cytoplasm
		Aminoacyl-tRNA-synthethases					
**79**	ZBAI_01417	Frs1	phenylalanyl-tRNA synthetase	2.3	2.59E-03	1.01E-02	Cytoplasm
		Protein folding and stabilization					
**1**	ZBAI_07617	Hsp60	Mitochondrial chaperonin	0.1	8.66E-05	2.52E-03	Mitochondrion
**23**	ZBAI_ 01079			0.3	7.44E-04	6.21E-03	
**11**	ZBAI_ 01501	Ssc1	Hsp70 family ATPase	0.2	5.30E-04	5.50E-03	Mitochondrion
**28**	ZBAI_ 01501			0.3	1.24E-03	7.13E-03	
**76**	ZBAI_05766			0.4	8.58E-03	2.23E-02	
**33**	ZBAI_05212	Ssb1	Hsp70 family ATPase	3.3	1.71E-03	8.42E-03	Cytoplasm, Plasma membrane
**61**	ZBAI_05212			2.7	1.56E-02	3.29E-02	
**38**	ZBAI_09562	Ssa1	Hsp70 family ATPase	3.2	1.23E-03	7.13E-03	Cytoplasm, Nucleus, Plasma membrane
**39**	ZBAI_09562			3.1	7.94E-03	2.23E-02	

**130**	ZBAI_01757			2	3.10E-03	1.15E-02	
**67**	ZBAI_ 06686	Hsc82	Cytoplasmic chaperone of the Hsp90 family	0.4	2.16E-04	3.70E-03	Cytoplasm, Mitochondrion, Plasma membrane
**65**	ZBAI_03386	Hsp26	Small heat shock protein	2.6	6.99E-04	6.17E-03	Cytoplasm, Nucleus
		Protein modification					
**161**	ZBAI_ 01634	Mas1	Smaller subunit of the mitochondrial processing protease	0.6	5.13E-03	1.56E-02	Mitochondrion
		Other					
**47**	ZBAI_02063	Ola1	P-loop ATPase identified as specifically interacting with the proteasome	0.3	1.45E-04	2.99E-03	Cytoplasm, Nucleus
		**Cellular transport**					
		Transporter routes					
**128**	ZBAI_07045	Gdi1	GDP dissociation inhibitor	2	1.36E-03	7.71E-03	Cytoplasm, Nucleus
**141**	ZBAI_00544	Kes1	Member of the yeast oxysterol binding protein family	0.6	8.02E-03	2.23E-02	Cytoplasm, Nucleus
		**Unclassified proteins**					
**147**	ZBAI_06262	Fmp41	Putative protein of unknown function	0.6	1.95E-02	3.73E-02	Mitochondrion
**153**	ZBAI_04092	Fmp12	Putative protein of unknown function	0.6	4.40E-03	1.46E-02	Mitochondrion
**99**	ZBAI_02189	Fsh1	Putative serine hydrolase	0.5	5.25E-03	1.56E-02	Cytoplasm, Nucleus
**149**	ZBAI_03069	YIL108W	Putative metalloendopeptidase	1.8	9.34E-03	1.62E-02	Cytoplasm, Nucleus

Table S2. Proteins identified in this work. The ISA1307 strain database protein accession number [2], protein description and the respective spot number, indicated in figure 2A, are associated. For each protein, theoretical pI and molecular weight are indicated. Protein identification was obtained by MALDI-TOF/TOF. The identity (%) of each protein-encoding ORF with CLIB213^T genome was obtained by BLAST (http://blast.ncbi.nlm.nih.gov/Blast.cgi). The relative migration distance (R_f) of the protein standards and proteins were manually determined to estimate the experimental molecular weight of each protein.

Spot number	Proteins identified	Protein Description	Theoretical Mw (kDa)	Theoretical pI	Experimental Mw (kDa)	Mascot Score	N Matched peptides MS	N Matched peptides MS/MS	Closest organism
**1**	ZBAI_07617	probable Heat shock protein 60, mitochondrial	60781.6	5.2	60-80	996	35	12	CLIB (99%)
**2**	ZBAI_04020	probable Malate dehydrogenase, mitochondrial	35189.3	8.8	30-40	644	14	7	CLIB (99%)
**3**	ZBAI_05689	Serine hydroxymethyltransferase, cytosolic	52127.7	7.7	50-60	1300	50	15	CLIB (100%)
**4**	ZBAI_09271	probable HXK2 - Hexokinase II	53820.9	5.9	50-60	533	24	11	CLIB (99%)
**5**	ZBAI_04020	probable Malate dehydrogenase, mitochondrial	35189.3	8.8	30-40	1020	20	10	CLIB (99%)
**6**	ZBAI_09271	probable HXK2 - Hexokinase II	53820.9	5.9	50-60	536	25	10	CLIB (99%)
**7**	ZBAI_01751	probable Ketol-acid reductoisomerase, mitochondrial	51783.8	6.9	≈40	1400	33	12	Other (96%)
**8**	ZBAI_08766	5-methyltetrahydropteroyltriglutamate--homocysteine methyltransferase	86202.6	6.7	80-110	1090	40	13	CLIB (100%)
**9**	ZBAI_08296	probable 37S ribosomal protein S26, mitochondrial	29838.9	7.1	20-30(≈30)	454	7	4	CLIB (99%)

**10**	ZBAI_01779	probable protein MET17	48499.8	5.6	≈50	971	21	10	Other (96%)
**11**	ZBAI_01501	Heat shock protein SSC1, mitochondrial	69623.1	5.6	60-80	1390	44	14	CLIB (99%)
**12**	ZBAI_01777	Aconitate hydratase, mitochondrial	85307.4	8.0	60-80(≈70)	118	11	2	Other (97%)
**13**	ZBAI_06465	Bifunctional purine biosynthesis protein ADE17	65275.7	8.0	60-80	1180	39	13	CLIB (100%)
**14**	ZBAI_06465	Bifunctional purine biosynthesis protein ADE17	65275.7	8.0	60-80	1270	43	14	CLIB (100%)
**15**	ZBAI_03268	probable Inositol-3-phosphate synthase	59578.4	5.3	≈60	360	24	5	Other (96%)
**17**	ZBAI_01038	Pyruvate decarboxylase	53587.3	5.2	≈60	514	21	5	Other (98%)
**18**	ZBAI_01038	probable ATP synthase subunit beta, mitochondrial	53587.3	5.2	50-60	1090	20	11	Other (98%)
**19**	ZBAI_09166	Malate synthase 1, glyoxysomal	62481.9	7.0	≈60	487	27	8	Other (96%)
**20**	ZBAI_05673	Actin	45305.9	5.6	40-50	820	27	12	CLIB (100%)
**21**	ZBAI_01422	Serine hydroxymethyltransferase, cytosolic	52141.7	7.7	50-60	923	44	13	Other (97%)
**23**	ZBAI_01079	probable Heat shock protein 60, mitochondrial	60779.6	5.2	60-80	830	35	10	Other (95%)
**24**	ZBAI_01179	probable Sulfate adenylyltransferase	57200.3	6.4	50-60	1000	36	12	CLIB (100%)
**25**	ZBAI_07987	Inorganic pyrophosphatase	32391.5	5.2	30-40	1180	20	12	CLIB (99%)
**26**	ZBAI_00097	probable NADPH-dependent alpha-keto amide reductase	35729.6	6.6	30-40	864	26	9	CLIB (99%)
**27**	ZBAI_02477	probable Peroxiredoxin type-2	18613.3	6.4	<20	527	11	4	Other (96%)
**28**	ZBAI_01501	Heat shock protein SSC1, mitochondrial	69623.1	5.6	50-60	107	7	2	CLIB (99%)
**29**	ZBAI_04020	probable Malate dehydrogenase, mitochondrial	35189.3	8.8	30-40	168	7	3	CLIB (99%)
**30**	ZBAI_01262	probable protein BMH1	35716.2	5.3	30-40	662	13	9	Other (98%)
**31**	ZBAI_03405	Phosphoglycerate kinase	44584.5	8.7	40-50	1600	37	13	CLIB (99%)
**32**	ZBAI_04207	probable ENO1 - Enolase I (2-phosphoglycerate dehydratase)	58021.0	6.4	40-50	1030	26	11	Other (97%)
**33**	ZBAI_05212	Heat shock protein SSB	71541.1	5.2	60-80	1160	26	11	Other (98%)

**34**	ZBAI_06832	probable Gamma-glutamyl phosphate reductase	49659.4	5.8	50-60	382	15	4	CLIB (99%)
**35**	ZBAI_08072	probable Delta-1-pyrroline-5-carboxylate dehydrogenase, mitochondrial	64084.5	7.6	50-60	491	32	7	CLIB (99%)
**36**	ZBAI_01751	probable Ketol-acid reductoisomerase, mitochondrial	51783.8	6.9	≈40	714	29	10	Other (96%)
**37**	ZBAI_08418	Citrate synthase, mitochondrial	52402.1	9.0	40-50	1280	33	15	Other (96%)
**38**	ZBAI_09562	Heat shock protein SSA2	69513.0	5.0	≈40	1120	27	12	CLIB (99%)
**39**	ZBAI_09562	Heat shock protein SSA2	69513.0	5.0	60-80	1230	33	13	CLIB (99%)
**41**	ZBAI_01779	probable protein MET17	48499.8	5.6	≈50	680	13	8	Other (96%)
**42**	ZBAI_01036	40S ribosomal protein S5	25908.6	6.8	≈30	662	20	9	CLIB (99%)
**43**	ZBAI_03278	probable COR1 - Ubiquinol--cytochrome-c reductase 44K core protein	48740.8	6.2	40-50	1120	24	11	Other (96%)
**44**	ZBAI_04908	Bifunctional purine biosynthesis protein ADE17	65251.5	6.7	60-80	1150	41	13	Other (95%)
**45**	ZBAI_05562	probable Transketolase 1	74236.3	6.2	60-80	725	25	11	CLIB (100%)
**46**	ZBAI_04020	probable Malate dehydrogenase, mitochondrial	35189.3	8.8	30-40	394	10	5	CLIB (99%)
**47**	ZBAI_02063	probable GTP-binding protein	43917.8	6.3	40-50	983	22	11	Other (98%)
**48**	ZBAI_03803	Elongation factor 2	93291.4	6.3	110-160 (≈110)	977	44	13	Other (96%)
**49**	ZBAI_08285	probable COR1 - Ubiquinol--cytochrome-c reductase 44K core protein	48654.7	6.2	40-50	1360	27	14	CLIB (100%)
**50**	ZBAI_05562	probable Transketolase 1	74236.3	6.2	60-80	1150	33	13	CLIB (100%)
**51**	ZBAI_04106	related to Catabolite repression protein CAT5	32892.3	5.5	20-30	441	13	6	CLIB (100%)
**52**	ZBAI_02995	D-lactate dehydrogenase [cytochrome] 3	55185.4	6.8	50-60	408	23	6	Other (95%)
**54**	ZBAI_04388	probable Superoxide dismutase [Mn], mitochondrial	25251.5	8.7	≈20	680	19	7	Other (97%)

**55**	ZBAI_09271	probable HXK2 - Hexokinase II	53820.9	5.9	50-60	509	23	8	CLIB (99%)
**58**	ZBAI_01422	Serine hydroxymethyltransferase, cytosolic	52141.7	7.7	50-60	781	39	9	Other (97%)
**60**	ZBAI_07063	probable Aconitate hydratase, mitochondrial	92264.4	8.5	80-110	1150	46	14	CLIB (99%)
**61**	ZBAI_05212	Heat shock protein SSB	71541.1	5.2	60-80	781	23	8	Other (98%)
**62**	ZBAI_08009	Glycerol-3-phosphate dehydrogenase [NAD(+)] 2	42958.2	5.3	40-50	529	18	5	CLIB (99%)
**63**	ZBAI_08441	Isocitrate dehydrogenase [NADP] cytoplasmic	46645.8	7.1	40-50	722	24	7	CLIB (100%)
**64**	ZBAI_01038	probable ATP synthase subunit beta, mitochondrial	53587.3	5.2	50-60	987	22	10	Other (98%)
**65**	ZBAI_03386	probable HSP26 - Heat shock protein	23348.0	5.3	20-30(≈30)	591	11	8	CLIB (99%)
**66**	ZBAI_03468	UGP1 - UTP--glucose-1-phosphate uridylyltransferase	56251.4	7.7	50-60	941	34	12	Other (96%)
**67**	ZBAI_06686	probable ATP-dependent molecular chaperone HSC82	82123.4	4.9	30-40	725	29	10	CLIB (99%)
**68**	ZBAI_01036	40S ribosomal protein S5	25908.6	6.8	50-60	577	20	10	CLIB (99%)
**69**	ZBAI_01182	Glyceraldehyde-3-phosphate dehydrogenase	35742.8	6.5	≈30	943	31	12	CLIB (99%)
**70**	ZBAI_01182	Glyceraldehyde-3-phosphate dehydrogenase	35742.8	6.5	30-40	749	17	10	CLIB (99%)
**71**	ZBAI_04932	Pyruvate kinase	54828.7	6.6	30-40	827	21	12	Other (98%)
**72**	ZBAI_01085	Tryptophan synthase	76603.2	6	30-40	664	33	13	Other (96%)
**73**	ZBAI_08000	probable Phosphoribosylaminoimidazole-succinocarboxamide synthase	34813.9	5.8	≈80	158	12	3	CLIB (99%)
**74**	ZBAI_06232	Saccharopine dehydrogenase [NADP(+), L-glutamate-forming]	48861.3	5.6	30-40	691	22	9	CLIB (99%)
**76**	ZBAI_05766	Heat shock protein SSC1, mitochondrial	69623.1	5.6	30-40	1270	34	14	Other (96%)
**77**	ZBAI_08408	Phosphoglycerate kinase	44584.5	8.8	40-50	424	16	4	Other (97%)
**79**	ZBAI_01417	probable Phenylalanine--tRNA ligase beta subunit	66908.8	5.7	60-80(≈80)	117	7	4	Other (96%)

**80**	ZBAI_08499	probable Fructose-bisphosphate aldolase	39751.1	6.0	20-30	152	6	2	CLIB (100%)
**81**	ZBAI_07777	Phospho-2-dehydro-3-deoxyheptonate aldolase, tyrosine-inhibited	39917.2	6.3	30-40	744	19	8	CLIB (100%)
**82**	ZBAI_00799	probable Aromatic/aminoadipate aminotransferase 1	55173.9	5.8	50-60	516	14	7	CLIB (100%)
**83**	ZBAI_01577	S-adenosylmethionine synthase 1	41832.7	5.3	40-50	1290	33	14	CLIB (99%)
**84**	ZBAI_03035	probable Dihydrolipoyllysine-residue acetyltransferase component of pyruvate dehydrogenase complex, mitochondrial	49428.8	8.2	50-60	160	6	3	Other (97%)
**85**	ZBAI_00201	probable Cystathionine beta-synthase	55035.6	5.9	50-60	898	27	11	CLIB (100%)
**86**	ZBAI_04207	probable ENO1 - Enolase I (2-phosphoglycerate dehydratase)	58021.0	6.4	≈40	428	10	8	Other (97%)
**87**	ZBAI_04053	probable Branched-chain-amino-acid aminotransferase, mitochondrial	44514.6	9.1	≈40	498	18	6	Other (96%)
**89**	ZBAI_07987	Inorganic pyrophosphatase	32391.5	5.2	30-40	792	11	8	CLIB (99%)
**90**	ZBAI_02973	probable Glycerol-3-phosphate dehydrogenase, mitochondrial	71131.7	6.8	30-40	523	25	6	CLIB (99%)
**91**	ZBAI_02973	probable Glycerol-3-phosphate dehydrogenase, mitochondrial	71131.7	6.8	30-40	552	25	8	CLIB (99%)
**92**	ZBAI_05708	probable Dihydrolipoyl dehydrogenase, mitochondrial	53412.5	7.1	50-60	1360	34	12	CLIB (100%)
**96**	ZBAI_01441	probable Dihydrolipoyl dehydrogenase, mitochondrial	53363.5	6.8	50-60	1580	35	14	Other (96%)
**97**	ZBAI_09663	Dihydroxy-acid dehydratase, mitochondrial	62580.8	7.6	≈60	716	30	8	CLIB (99%)
**98**	ZBAI_00399	Spermidine synthase	33239.1	5.5	30-40	470	12	5	Other (96%)
**99**	ZBAI_02189	probable Family of serine hydrolases 1	26143.8	5.7	20-30	639	19	7	Other (95%)
**100**	ZBAI_03696	Peroxiredoxin TSA1	21614.8	5.6	≈20	941	11	8	Other (97%)
**102**	ZBAI_02355	probable Alcohol dehydrogenase 1	43707.4	8.0	40-50	1130	24	13	CLIB (100%)
**103**	ZBAI_07717	ENO1 - Enolase I (2-phosphoglycerate dehydratase)	46681.2	6.3	40-50	588	20	8	CLIB (99%)

**107**	ZBAI_02973	probable Glycerol-3-phosphate dehydrogenase, mitochondrial	71131.7	6.8	60-80	346	17	6	CLIB (99%)
**113**	ZBAI_02009	probable Acetolactate synthase catalytic subunit, mitochondrial	74796.8	7.3	60-80	573	31	9	Other (96%)
**114**	ZBAI_08717	probable Dihydrolipoyllysine-residue acetyltransferase component of pyruvate dehydrogenase complex, mitochondrial	49329.6	7.6	50-60(≈60)	400	12	4	CLIB (100%)
**115**	ZBAI_01777	Aconitate hydratase, mitochondrial	85307.4	8.0	80-110	983	35	14	Other (97%)
**116**	ZBAI_09839	probable ATP synthase subunit d, mitochondrial	19465.4	7.8	≈20	521	11	6	Other (98%)
**120**	ZBAI_03405	Phosphoglycerate kinase	44584.5	8.7	40-50	890	18	11	CLIB (99%)
**121**	ZBAI_00026	Adenylosuccinate synthetase	48249.4	6.8	40-50	714	13	7	CLIB (100%)
**122**	ZBAI_08357	probable RIP1 - Ubiquinol--cytochrome-c reductase iron-sulfur protein precursor	23234.8	7.7	20-30	599	11	6	Other (95%)
**123**	ZBAI_04932	Pyruvate kinase	54828.7	6.6	≈60	1020	28	14	Other (98%)
**124**	ZBAI_08285	probable COR1 - Ubiquinol--cytochrome-c reductase 44K core protein	48654.7	6.2	40-50(≈50)	519	18	7	CLIB (100%)
**125**	ZBAI_03351	probable RIP1 - Ubiquinol--cytochrome-c reductase iron-sulfur protein precursor	23217.8	8.3	20-30	326	9	4	CLIB (100%)
**126**	ZBAI_00026	Adenylosuccinate synthetase	48249.4	6.8	40-50	384	14	4	CLIB (100%)
**127**	ZBAI_03351	probable RIP1 - Ubiquinol--cytochrome-c reductase iron-sulfur protein precursor	23217.8	8.3	20-30	466	10	4	CLIB (100%)
**128**	ZBAI_07045	Rab GDP-dissociation inhibitor	50930.8	5.4	≈50	255	11	5	CLIB (100%)
**129**	ZBAI_08742	probable POR1 - Voltage dependent anion-selective channel (YVDAC1) of the mitochondrial outer membrane	29313.3	7.8	20-30	1010	26	9	CLIB (99%)
**130**	ZBAI_01757	Heat shock protein SSA2	69512.0	5.0	40-50	497	17	10	Other (95%)

**131**	ZBAI_07717	ENO1 - Enolase I (2-phosphoglycerate dehydratase)	46681.2	6.3	≈40	77	7	2	CLIB (99%)
**132**	ZBAI_08742	probable POR1 - Voltage dependent anion-selective channel (YVDAC1) of the mitochondrial outer membrane	29313.3	7.8	20-30	13	7	5	CLIB (99%)
**133**	ZBAI_00269	Homoisocitrate dehydrogenase, mitochondrial	39846.9	8.7	≈40	799	24	9	CLIB (100%)
**134**	ZBAI_03500	probable Fructose-bisphosphate aldolase	39685.0	6.0	≈40	643	15	6	Other (97%)
**135**	ZBAI_04908	Bifunctional purine biosynthesis protein ADE17	65251.5	6.7	60-80	747	23	10	Other (96%)
**136**	ZBAI_03414	Citrate synthase, mitochondrial	52406.2	9.0	≈50	858	22	12	CLIB (99%)
**137**	ZBAI_04734	probable Adenylate kinase 1	24651.3	7.0	20-30	771	28	9	Other (96%)
**139**	ZBAI_03097	probable Threonine dehydratase, mitochondrial	63459.8	7.4	50-60	1090	27	13	Other (96%)
**140**	ZBAI_04908	Bifunctional purine biosynthesis protein ADE17	65251.5	6.7	60-80	421	23	8	Other (95%)
**141**	ZBAI_00544	probable protein KES1	48557.7	5.9	≈50	288	8	4	Other (97%)
**142**	ZBAI_06765	probable Potassium-activated aldehyde dehydrogenase, mitochondrial	56318.1	5.8	≈60	646	14	8	CLIB (99%)
**143**	ZBAI_00484	probable Fumarate reductase	53962.9	8.1	50-60	1140	25	14	CLIB (100%)
**144**	ZBAI_00484	probable Fumarate reductase	53962.9	8.1	50-60	1010	17	10	CLIB (100%)
**145**	ZBAI_05673	Actin	45305.9	5.6	40-50	520	14	9	CLIB (100%)
**146**	ZBAI_08742	probable POR1 - Voltage dependent anion-selective channel (YVDAC1) of the mitochondrial outer membrane	29313.3	7.8	20-30	394	7	4	CLIB (99%)
**147**	ZBAI_06262	probable FMP41 - Found in Mitochondrial Proteome	29255.8	7.1	≈30	363	9	5	CLIB (100%)
**148**	ZBAI_00463	Probable aconitate hydratase 2	85795.7	6.2	≈110(>)	738	31	9	Other (95%)
**149**	ZBAI_03069	related to zinc metalloproteinase YIL108W	67188.9	7.2	≈80	202	13	4	Other (96%)

**150**	ZBAI_05994	probable Dihydrolipoyllysine-residue succinyltransferase component of 2-oxoglutarate dehydrogenase complex, mitochondrial	49383.9	9.2	50-60	422	19	10	Other (95%)
**151**	ZBAI_00412	Elongation factor 1-alpha	50049.8	9.2	30-40	903	22	12	Other (98%)
**152**	ZBAI_02050	probable Alcohol dehydrogenase 4, mitochondrial	39727.5	7.1	≈40	923	19	9	Other (96%)
**153**	ZBAI_04092	related to oxidoreductase AIM17	52428.3	6.1	40-50(≈50)	482	20	6	Other (95%)
**154**	ZBAI_05617	Probable aconitate hydratase 2	85843.7	6.2	≈110 (>)	805	30	10	CLIB (99%)
**155**	ZBAI_02835	Triosephosphate isomerase	27027.9	7.7	20-30	332	12	7	CLIB (100%)
**156**	ZBAI_03797	probable Isocitrate dehydrogenase [NAD] subunit 2, mitochondrial	39623.7	9.2	≈40	808	13	9	Other (97%)
**157**	ZBAI_05297	probable protein ARG5,6, mitochondrial	94938.0	6.9	≈40	622	19	6	Other (95%)
**158**	ZBAI_03439	probable Acetolactate synthase small subunit, mitochondrial	34519.6	6.2	30-40	760	13	9	Other (94%)
**159**	ZBAI_00128	probable protein ARG5,6, mitochondrial	94965.0	7.2	≈40	282	10	3	CLIB (99%)
**160**	ZBAI_03803	Elongation factor 2	93291.4	6.3	≈110 (>)	395	15	7	Other (96%)
**161**	ZBAI_01634	probable Mitochondrial-processing peptidase subunit beta	51461.6	7.8	≈50	388	15	6	CLIB (99%)
**162**	ZBAI_04053	probable Branched-chain-amino-acid aminotransferase, mitochondrial	44514.6	9.1	≈40	1250	27	13	Other (96%)
**163**	ZBAI_01905	probable Pyruvate dehydrogenase E1 component subunit beta, mitochondrial	39255.1	5.2	30-40	674	13	8	Other (96%)
**165**	ZBAI_09676	probable Flavohemoprotein	44415.9	5.7	40-50(≈40)	839	21	11	CLIB (99%)
**166**	ZBAI_06025	probable Mitochondrial peroxiredoxin PRX1	27983.1	8.9	20-30	572	18	9	Other (95%)

Supplemental Data References:

1. Jensen LJ, Kuhn M, Stark M, Chaffron S, Creevey C, Muller J, Doerks T, Julien P, Roth A, Simonovic M, Bork P, von Mering C (**2009**). STRING 8-a global view on proteins and their functional interactions in 630 organisms. **Nucleic Acids Res** 37: D412-D416. doi: Doi 10.1093/Nar/Gkn760.

2. Mira NP, Munsterkotter M, Dias-Valada F, Santos J, Palma M, Roque FC, Guerreiro JF, Rodrigues F, Sousa MJ, Leão C, Guldener U, Sá-Correia I (**2014**). The Genome Sequence of the Highly Acetic Acid-Tolerant *Zygosaccharomyces bailii*-Derived Interspecies Hybrid Strain ISA1307, Isolated From a Sparkling Wine Plant. **DNA Res** 21(3): 299-313. doi: 10.1093/dnares/dst058.
